# Comparative Evaluation of the Early Immunological Effects of a Novel Piperidine α-Hydroxyphosphonate and a Phosphorodiamidate Morpholino Oligomer in an Imiquimod-Induced Psoriasis Model

**DOI:** 10.3390/molecules31213926

**Published:** 2026-11-01

**Authors:** Marina K. Balabekova, Tolganay Y. Zharkynbek, Meruyert B. Mussilim, Zhansaya Nuralikyzy, Gulgul K. Kairanbayeva, Tulegen M. Seilkhanov, Altynay B. Kaldybayeva, Valentina K. Yu, Mariya Li, Milana A. Alimova, Elmira M. Satbayeva, Anar Kh. Balapanova, Sulev Koks

**Affiliations:** 1 Department of Pathological Physiology Named After Professor A.N. Nurmukhambetov, Asfendiyarov Kazakh National Medical University, 94 Tole-bi St., Almaty 050000, Kazakhstan; balabekova.m@kaznmu.kz (M.K.B.); nuralikyzy.zh@kaznmu.kz (Z.N.); kairanbayeva.g@kaznmu.kz (G.K.K.); li.maria@kaznmu.kz (M.L.); alimova.m@kaznmu.kz (M.A.A.); 2 Laboratory of Synthetic and Natural Medicinal Compounds Chemistry, A.B. Bekturov Institute of Chemical Sciences, 106 Sh. Ualikhanov St., Almaty 050010, Kazakhstan; tolganay.zharkynbek@gmail.com (T.Y.Z.); altin_28.94@mail.ru (A.B.K.); 3 Laboratory of Engineering Profile NMR Spectroscopy, Sh. Ualikhanov Kokshetau University, 76 Abai St., Kokshetau 020000, Kazakhstan; tseilkhanov@mail.ru; 4 Department of Pharmacology, Asfendiyarov Kazakh National Medical University, 94 Tole-bi St., Almaty 050000, Kazakhstan; satbaeva.e@kaznmu.kz; 5 Department of Pathology, National Hospital of the Medical Center of the Administration of the President of the Republic of Kazakhstan, 139 Panfilov St., Almaty 050000, Kazakhstan; balapanova.an@kaznmu.kz; 6 Health Futures Institute, Murdoch University, 90 South St., Perth, WA 6150, Australia; sulev.koks@murdoch.edu.au

**Keywords:** psoriasis, imiquimod, α-hydroxyphosphonate, piperidine oxyphosphonate, OPP, PMO, T lymphocytes, IL-17A, IL-22, IL-10, TGF-β1

## Abstract

Psoriasis is a chronic immune-mediated inflammatory disease associated with dysregulation of T-cell responses and cytokine signaling. The aim of this study was to synthesize and structurally characterize a piperidine α-hydroxyphosphonate (OPP) and to comparatively evaluate the effects of OPP and the vivo-phosphorodiamidate morpholino oligomer (vPMO) in a preclinical model of imiquimod-induced psoriasis. OPP was synthesized by hydrophosphorylation of 1-(3-isopropoxypropyl)piperidin-4-one with dimethyl phosphite; its structure was confirmed by IR, NMR spectroscopy, and elemental analysis. Psoriasis was induced in rats by topical application of imiquimod (IMQ). The animals were randomly allocated into six groups: control (C), experimental psoriasis (Ps), vehicle-treated (Ps/V), glucocorticosteroid-treated (Ps/GCS), piperidine α-hydroxyphosphonate-treated (Ps/OPP), and vivo-phosphorodiamidate morpholino oligomer-treated (Ps/vPMO) groups. Clinical manifestations were assessed on Days 3, 5, and 7 using a modified Psoriasis Area and Severity Index (PASI). During the 7-day observation period, body weight and the modified Psoriasis Area and Severity Index (PASI) score were assessed on Days 3, 5, and 7. Histopathological changes were evaluated using hematoxylin and eosin (H&E) staining. On day 7, CD3^+^CD45R^−^, CD4^+^CD8^−^, and CD4^+^CD25^+^ T-cell subpopulations were determined by flow cytometry, while serum concentrations of IL-6, TNF-α, IL-10, TGF-β1, IL-17A, and IL-22 were measured using an enzyme-linked immunosorbent assay (ELISA). IMQ administration resulted in the progressive development of psoriasiform skin lesions characterized by hyperkeratosis, acanthosis, inflammatory infiltration of the dermis, body weight loss, decreased numbers of CD3^+^CD45R^−^ and CD4^+^CD8^−^ cells, increased levels of IL-6, IL-17A, and IL-22, and reduced IL-10 levels. OPP treatment was associated with a reduction in the total PASI score and the severity of scaling, as well as decreased serum concentrations of IL-6, IL-17A, and IL-22 and an increased IL-10 concentration. In addition, OPP increased the number of CD4^+^CD8^−^ cells compared with the psoriasis group, whereas the number of CD4^+^CD25^+^ cells remained below the control level. In contrast, vPMO treatment exhibited a distinct response profile characterized by increased numbers of CD3^+^CD45R^−^ and CD4^+^CD8^−^ cells, decreased IL-17A and IL-22 levels, increased IL-10 levels, and a particularly pronounced increase in TGF-β1 while producing less pronounced changes in certain cutaneous manifestations. Overall, OPP was characterized by a more consistent pattern of clinical, histopathological, and immunological changes, whereas vPMO treatment predominantly resulted in more pronounced alterations in systemic immunological parameters. These findings indicate distinct patterns of biological effects of OPP and vPMO in the IMQ-induced psoriasiform skin lesion model and support further preclinical investigation of these compounds. However, the observed changes do not allow definitive conclusions to be drawn regarding the underlying molecular mechanisms or the involvement of specific cellular pathways and should be interpreted in light of the limited observation period, the restricted panel of cellular markers examined, the absence of certain experimental controls, and the lack of pharmacokinetic data.

## 1. Introduction

Psoriasis is a chronic immune-mediated inflammatory disease involving complex interactions among immune cells, cytokines, and keratinocytes. Dysregulation of T-cell responses is accompanied by altered production of pro-inflammatory and regulatory mediators, including IL-6, TNF-α, IL-17A, IL-22, IL-10, and TGF-β1, contributing to sustained inflammation and the development of characteristic skin changes [1,2]. The current understanding of psoriasis emphasizes the involvement of both innate and adaptive immunity and the close interplay between immune and epidermal mechanisms [3,4].

The development of molecular approaches has expanded opportunities to target individual components of the pathological process. In addition to biologics and small molecules, gene-directed strategies, including antisense oligonucleotides, are receiving increasing attention [5,6]. In particular, antisense oligonucleotides targeting IRF4 have reduced manifestations of IMQ-induced psoriasis and the number of IL-17A-producing CD4^+^ cells [7]. At the same time, inadequate or lost treatment responses, the need for long-term therapy, and differences in clinical profiles continue to drive the search for new approaches [8,9]. The possibility of combining agents with different mechanisms of action is also under investigation [3,10,11].

In Kazakhstan, the synthesis and experimental investigation of piperidine and related compounds has been developed systematically. We have previously investigated these compounds in models of aseptic inflammation and acute lung injury, assessing cellular composition, T-lymphocyte subpopulations, cytokine profiles, and reparative processes [12,13,14,15]. These studies provided an experimental basis for extending this investigation to new structural classes of piperidine compounds and new models of immune-mediated inflammatory pathology.

The OPP investigated in the present study is dimethyl [4-hydroxy-1-(3-isopropoxypropyl)piperidin-4-yl]phosphonate, an α-hydroxyphosphonate combining a tertiary piperidine nitrogen atom, phosphoryl and hydroxyl groups, two methoxy groups, and a flexible ether-containing N-substituent. α-Hydroxyphosphonates are regarded as a chemically and biologically important class of compounds [16,17]. The presence of multiple heteroatom-containing centers provides the potential for a variety of intermolecular interactions, but in itself does not determine a specific pharmacological target.

Previous studies of related piperidine oxyphosphonates have demonstrated the ability of this structural class to form complexes with Pd(II), Pt(II), and Cu(II), as well as a dependence of their functional properties on the nature of the heteroatoms present [18,19,20]. These findings confirm the chemical functionality of the oxyphosphonate scaffold but do not provide direct evidence for the mechanism of action of OPP in psoriasis. Therefore, in the present study, the structure of the compound is discussed only in relation to the observed phenotypic and immunological profile.

The second compound investigated in the present study was vPMO, targeting IL1RL2. For vPMO, the present study forms part of a broader program, involving sequential study of immunological, morphological and transcriptomic effects. Comparison of chemically distinct entities—a small-molecule OPP and a gene-directed vPMO—allows for identification of shared and divergent profiles of early cellular and humoral changes without assuming a priori that they have identical mechanisms of action.

The aim of the present study was to synthesize and structurally characterize OPP and to comparatively evaluate the clinical and early immunological changes associated with topical treatment with OPP and vPMO in an IMQ-induced psoriasis model. Changes in skin manifestations and body weight, T-lymphocyte subpopulations, and serum concentrations of IL-6, TNF-α, IL-10, TGF-β1, IL-17A, and IL-22 were assessed.

## 2. Results

### 2.1. Synthesis and Structural Characterization of Dimethyl [4-Hydroxy-1-(3-Isopropoxypropyl)piperidin-4-yl]phosphonate (OPP)

**Scheme 1:**
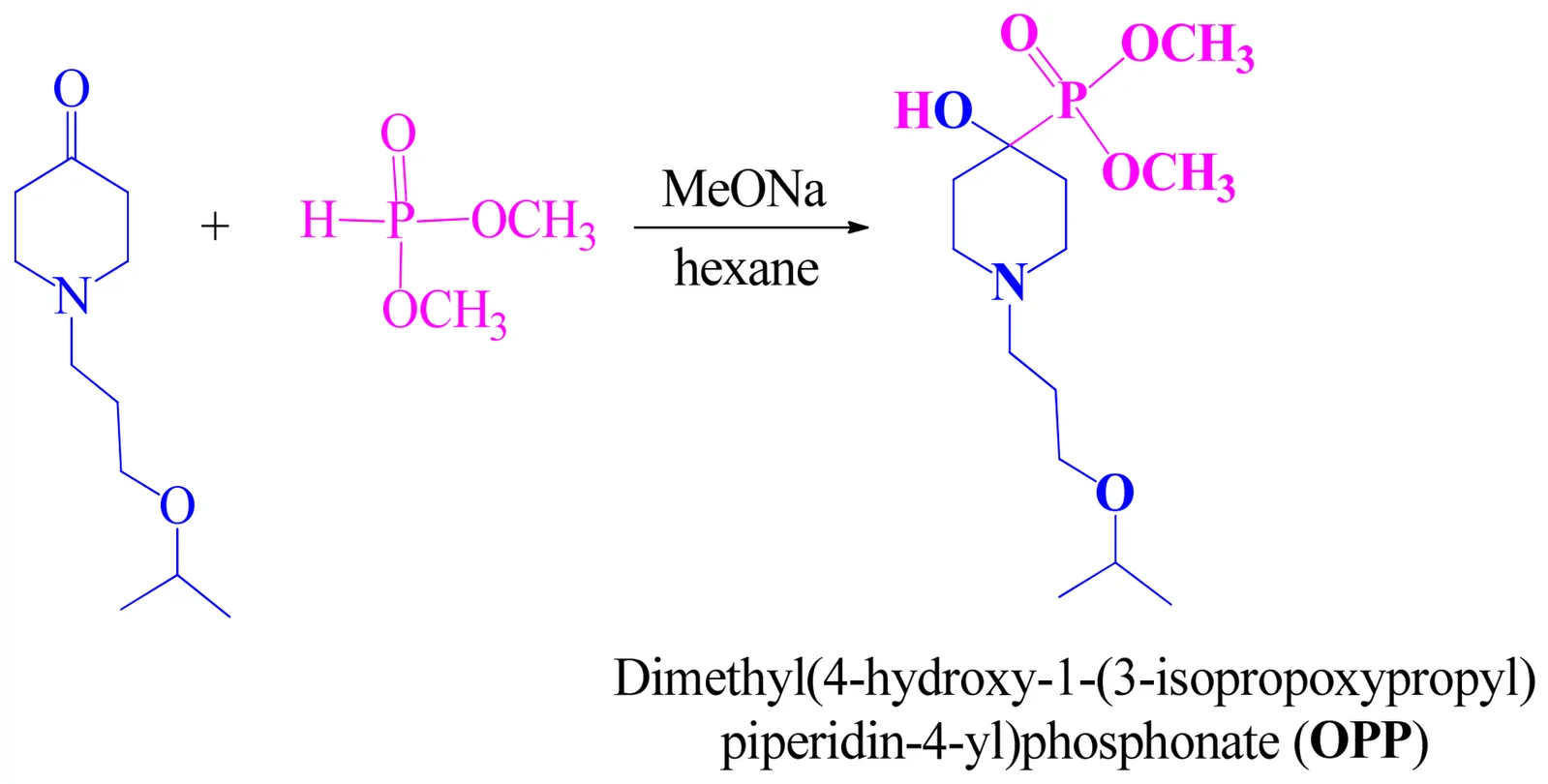
Synthesis of dimethyl [4-hydroxy-1-(3-isopropoxypropyl)piperidin-4-yl]phosphonate (OPP).

The target oxyphosphonate (OPP) was synthesized by hydrophosphorylation of the carbonyl group of 1-(3-isopropoxypropyl)piperidin-4-one [16] with dimethyl phosphite in the presence of sodium methoxide (Scheme 1). This transformation represents the base-catalyzed addition of dialkyl phosphites to aldehydes and ketones via the Pudovik–Abramov reaction, one of the most straightforward approaches to α-hydroxyphosphonates [17,18]. The use of 1.5 equivalents of dimethyl phosphite and controlled addition of the catalyst ensured complete consumption of the starting ketone, as monitored by TLC. Following low-temperature crystallization and recrystallization from hexane, OPP was isolated in 70% yield as a crystalline product with a melting point of 72–73 °C.

In the presence of sodium methoxide, the nucleophilicity of the phosphorus-containing reagent is enhanced, followed by nucleophilic attack at the carbonyl carbon of piperidin-4-one and subsequent protonation of the resulting alkoxide. As a result, the C–P bond and the hydroxyl group at C-4 are formed in a single step. The precipitation observed upon addition of the catalyst, followed by conversion into an oily phase, likely reflects changes in the solubility of the product and intermediate ionic species in the nonpolar medium. Subsequent cooling to −23 °C promotes crystallization of the final oxyphosphonate.

The IR spectrum (Figure S1) is consistent with the conversion of the ketone into the corresponding α-hydroxyphosphonate. The broad band at 3319 cm^−1^ is assigned to O–H stretching vibrations, while the intense band at 1222 cm^−1^ corresponds to P=O stretching. The bands observed at 1179, 1079, and 1039 cm^−1^ are attributed to the P–OCH_3_ and C–O–C moieties. The absence of an intense ketone absorption band in the characteristic ν(C=O) region further supports the consumption of the starting piperidin-4-one.

The ^1^H NMR spectrum (Figure S2) contains the full set of signals expected for the N-(3-isopropoxypropyl), piperidine, and dimethyl phosphonate moieties. The integrated signal intensities account for all 28 protons of the molecule. The six-proton signal at δ 1.00–1.07 ppm is assigned to the two methyl groups of the isopropoxy moiety, the NCH_2_CH_2_CH_2_O and OCH(CH_3_)_2_ protons resonate within the δ 1.60–3.40 ppm region, and the six protons of the P(OCH_3_)_2_ group appear at δ 3.51–3.74 ppm. The hydroxyl proton signal is observed at δ 3.03–3.09 ppm.

The ^13^C NMR spectrum (Figure S3) displays nine groups of resonances, consistent with the symmetry of the piperidine ring and the equivalence of the C-2/C-6 and C-3/C-5 pairs, and of the methoxy and isopropyl methyl carbon atoms. The most diagnostic feature is the C-4 resonance, observed as a doublet centered at δ(C) 69.17 ppm (^1^J(C,P) = 168.9 Hz; components at 68.33 and 70.01 ppm), supporting the presence of a direct C-4–P bond and the formation of the α-hydroxyphosphonate center.

The two-dimensional NMR spectra (Figures S4–S6) further confirm the connectivity of the molecular fragments. In the COSY spectrum, H-7/H-8 and H-8/H-9 correlations are observed for the NCH_2_CH_2_CH_2_O chain, together with H-11/H-12,17 correlations for the isopropoxy moiety. Direct H/C correlations in the HMQC spectrum assign H-7, H-8, H-9, and H-11 to C-7, C-8, C-9, and C-11, respectively. The long-range HMBC correlations H-7 → C-2,6 and H-9 → C-11 confirm attachment of the side chain to the nitrogen atom and the presence of the terminal isopropoxy group, whereas the H-2_eq_,6_eq_ → C-4 correlation is consistent with the location of the hydroxyphosphonate center at C-4. Taken together, the 1D and 2D NMR data exclude the alternative 3-methoxypropyl structural assignment.

The results of elemental analysis were consistent with the calculated values for C_13_H_28_NO_5_P. Thus, the isolated yield, physical characteristics, IR spectral data, elemental composition, and the combined ^1^H, ^13^C, COSY, HMQC, and HMBC NMR data consistently support the proposed structure of OPP. These findings extend the previously developed approach to N-substituted piperidin-4-yl oxyphosphonates [19,20,21].

### 2.2. Evaluation of the Effects of Imiquimod and Subsequent Treatment on Rat Body Weight and PASI Score

**Figure 1:**
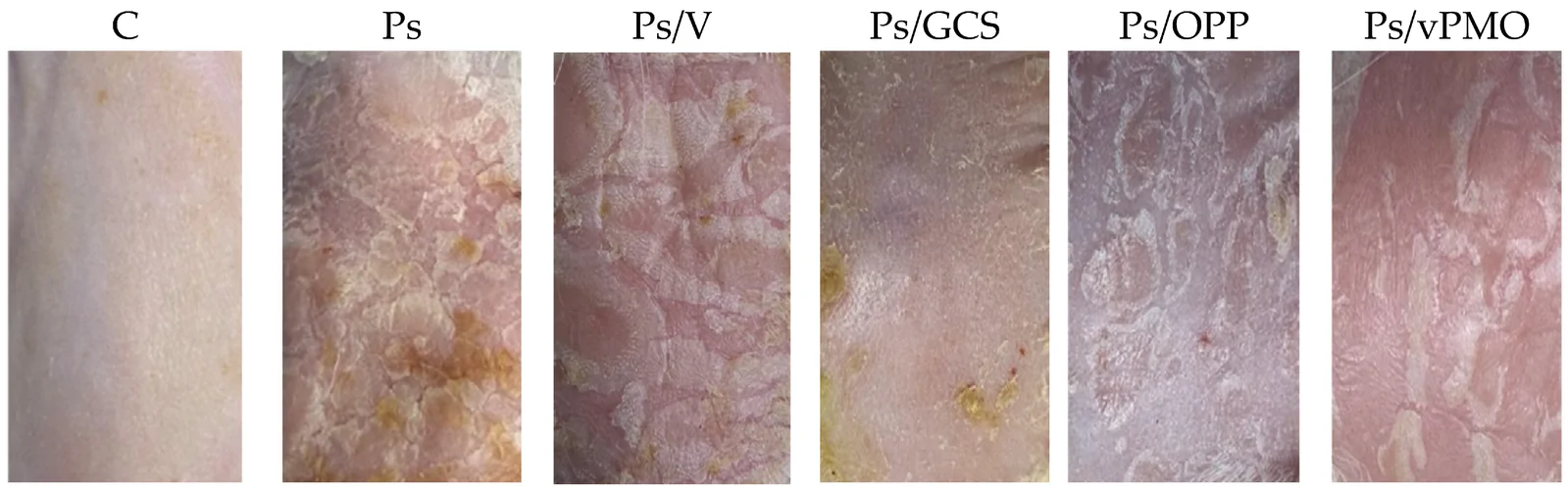
Representative macroscopic images of rat skin from the experimental groups: control, Ps, Ps/V, Ps/GCS, Ps/OPP, and Ps/vPMO, respectively.

Experimental psoriasis was induced by topical application of imiquimod (IMQ). Changes in PASI scores and body weight were assessed on Days 3, 5, and 7 and compared among the experimental groups. Macroscopic images of the dorsal skin from animals in all experimental groups are shown in Figure 1.

**Figure 2:**
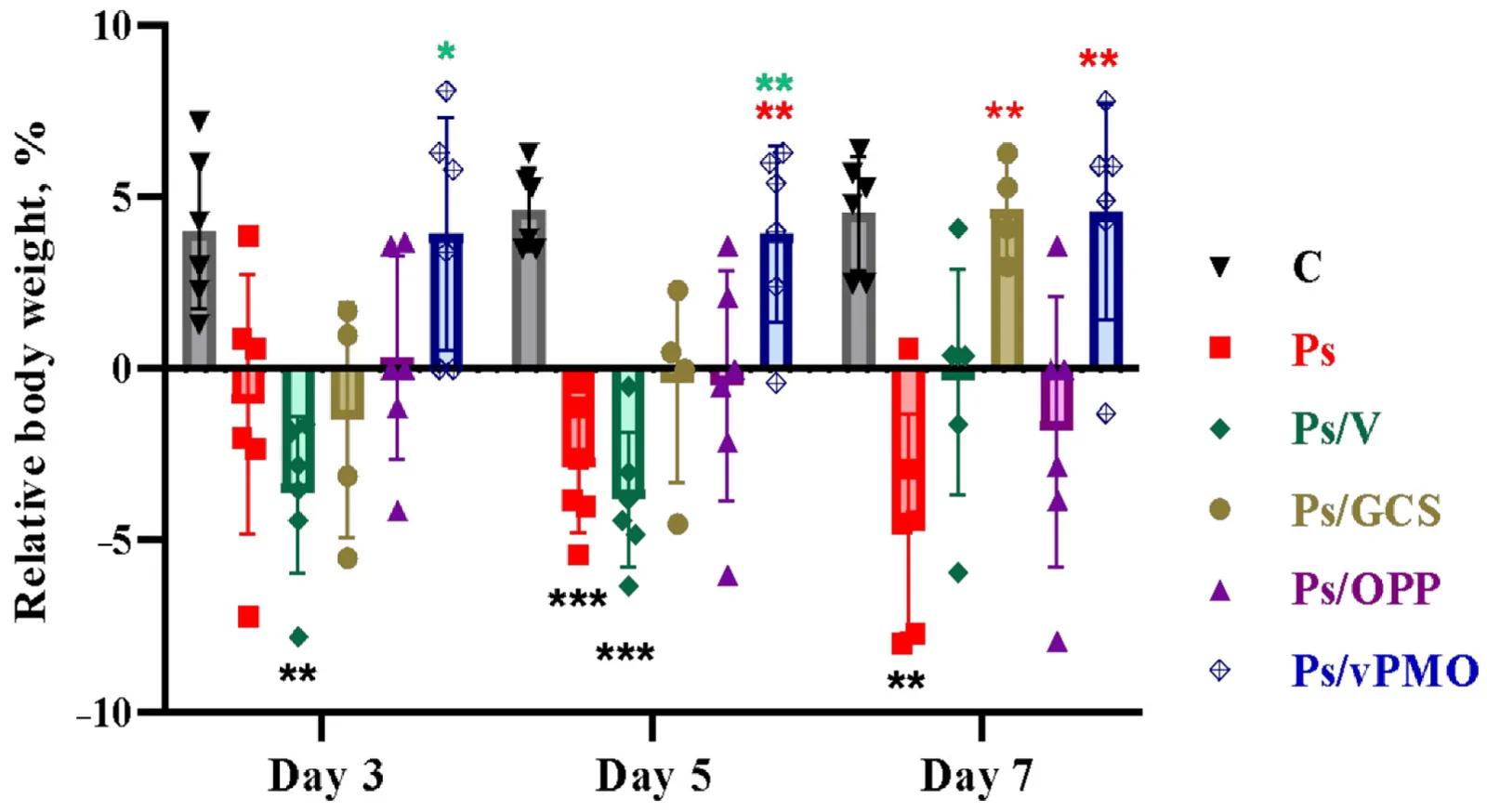
Effects of treatments on rats’ body weight. Statistical differences between groups over time were determined using two-way repeated-measures analysis of variance (ANOVA), followed by Tukey’s multiple comparison test: * vs control, * vs psoriasis, * vs Ps/V. The number of asterisks of the same color corresponded to the following *p*-values: * *p* ≤ 0.05, ** *p* ≤ 0.01, and *** *p* ≤ 0.001. All data are represented as mean ± SD. n = 6 rats per group.

On Day 3, a reduction in body weight relative to baseline was observed in the experimental psoriasis, vehicle-treated, and GCS-treated groups, whereas the control and vPMO-treated groups showed an increase in body weight. A statistically significant reduction in body weight was observed in the vehicle-treated group compared with the control and vPMO-treated groups (−3.6% ± 2.2% vs. 4.0% ± 2.2% and 3.9% ± 3.3% vs. 4.0% ± 2.2%, respectively; *p* ≤ 0.01 and *p* ≤ 0.05). On Day 5, the observed pattern was generally maintained. The experimental psoriasis and vehicle-treated groups showed significant reductions in body weight compared with the control group (−2.8% ± 1.8% and −3.8% ± 1.9% vs. 4.6% ± 1.2%, respectively; *p* ≤ 0.001 for both comparisons). In contrast, the vPMO-treated group showed a significant increase in body weight compared with both the experimental psoriasis and vehicle-treated groups (3.9% ± 2.5% vs. −2.8% ± 1.8% and −3.8% ± 1.9%, respectively; *p* ≤ 0.01 for both comparisons). By Day 7, body weight was significantly reduced relative to baseline in the experimental psoriasis group compared with the control, GCS-treated, and vPMO-treated groups (−4.4% ± 3.1% vs. 4.5% ± 1.6%, 4.6% ± 1.4%, and 4.5% ± 3.1%, respectively; *p* ≤ 0.01 for all comparisons) (Figure 2).

Overall, a progressive reduction in body weight was observed in the untreated experimental psoriasis group following IMQ administration, whereas body weight increased in the control group. This pattern may reflect a systemic effect of IMQ-induced inflammation beyond the local skin manifestations of IMQ-induced inflammatory skin changes.

The severity of psoriasis-like skin lesions was assessed using a modified PASI score. Erythema, scaling, and skin thickening on the dorsal surface of the rats were evaluated by an independent observer who was blinded to the treatment allocation. Each parameter was scored on a 0–4 scale, with 0 representing no lesions, 1 mild, 2 moderate, 3 severe, and 4 very severe lesions. The individual scores were summed to obtain a total PASI score ranging from 0 to 12, with higher scores indicating greater severity of skin inflammation.

**Figure 3:**
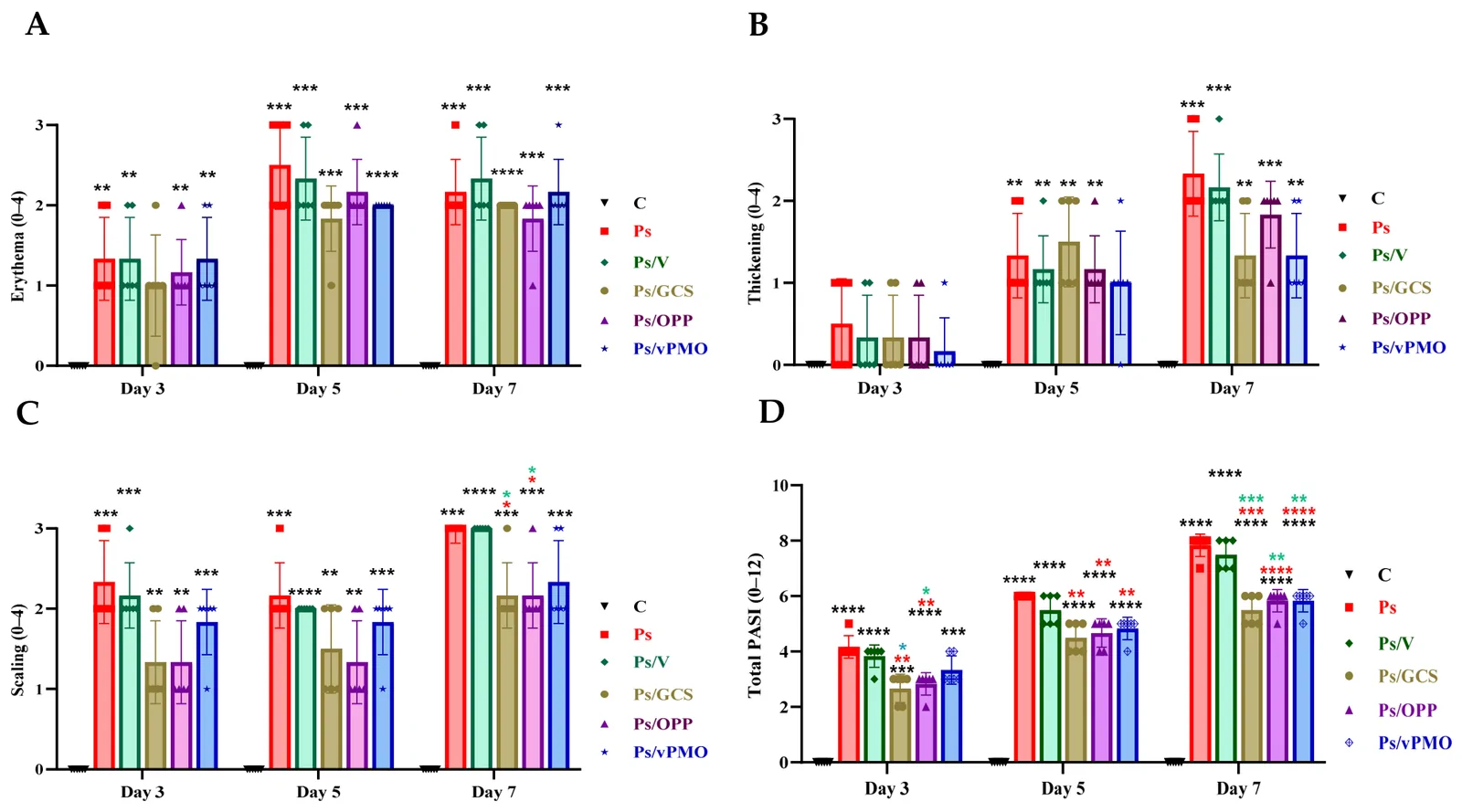
Dynamics of clinical manifestations of psoriasis over 7 days: (**A**) erythema; (**B**) scaling; (**C**) skin thickening; (**D**) total modified PASI score. Statistical differences between groups over time were determined using two-way repeated-measures analysis of variance (ANOVA), followed by Tukey’s multiple comparison test: * vs control, * vs psoriasis, * vs Ps/V. The number of asterisks of the same color corresponded to the following *p*-values: * *p* ≤ 0.05, ** *p* ≤ 0.01, *** *p* ≤ 0.001, and **** *p* ≤ 0.0001. All data are represented as mean ± SD. n = 6 rats per group.

No erythema, scaling, or skin thickening was observed in the control group throughout the observation period; accordingly, the scores for each individual clinical manifestation and the total PASI score remained at 0 at all assessment time points. In the Ps group, the severity of all three clinical manifestations increased progressively during the observation period, with the most pronounced changes observed on Days 5 and 7. Erythema reached a maximum score of 2.5 ± 0.5 on Day 5, whereas scaling and skin thickening reached maximum scores of 3.3 ± 0.5 and 2.3 ± 0.5, respectively, on Day 7 (Figure 3A–C). Consequently, the total PASI score increased progressively and reached its maximum value on Day 7 (7.8 ± 0.4) (Figure 3D). No significant differences were observed between the Ps/V and the Ps groups in any of the assessed clinical parameters, including erythema, scaling, and skin thickening, at any assessment time point. Likewise, no significant difference was observed in the total PASI score between these groups (Figure 3D). OPP and GCS did not produce a significant reduction in erythema and skin thickening scores at any assessment time point but, on Day 7, both OPP and GCS significantly reduced scaling compared with the Ps group (2.1 ± 0.4 vs. 3.3 ± 0.5; *p* ≤ 0.5 for both comparisons) (Figure 3C). Despite the limited effects on individual PASI components, significant differences in the total PASI score were observed between the treatment and Ps groups at several assessment time points. On Day 3, the total PASI score was significantly lower in the Ps/OPP and Ps/GCS groups than in the Ps group (2.8 ± 0.4, and 2.6 ± 0.5 vs. 4.1 ± 0.4, respectively; *p* ≤ 0.01 for both comparisons). On Day 5, the corresponding total PASI scores were 4.6 ± 0.5, and 4.5 ± 0.5 in the Ps/OPP, and Ps/GCS groups, respectively, compared with 6.0 ± 0.0 in the Ps group (*p* ≤ 0.01 for both comparisons). By Day 7, the total PASI score remained significantly lower in the Ps/OPP, and Ps/GCS groups than in the Ps group, with scores of 5.8 ± 0.4, and 5.5 ± 0.5, respectively, compared with 7.8 ± 0.4 (*p* ≤ 0.0001 for all comparisons). vPMO treatment did not reduce any of the individual clinical manifestations. Nevertheless, it resulted in a significant reduction in the total PASI score on Days 5 (4.8 ± 0.4 vs. 6.0 ± 0, *p* ≤ 0.05) and 7 (5.8 ± 0.4 vs. 7.8 ± 0.4, *p* ≤ 0.001) compared with the Ps group (Figure 3D).

Overall, imiquimod induced a progressive increase in the severity of psoriasis-like skin lesions, as reflected by the increase in the total PASI score over the observation period. Compared with the experimental psoriasis group, lower total PASI scores were observed in the OPP-, GCS-, and vPMO-treated groups at selected time points, whereas differences in individual PASI components were limited. These findings indicate differences in the clinical severity of skin lesions among the treatment groups during the observation period.

### 2.3. Histological Analysis

**Figure 4:**
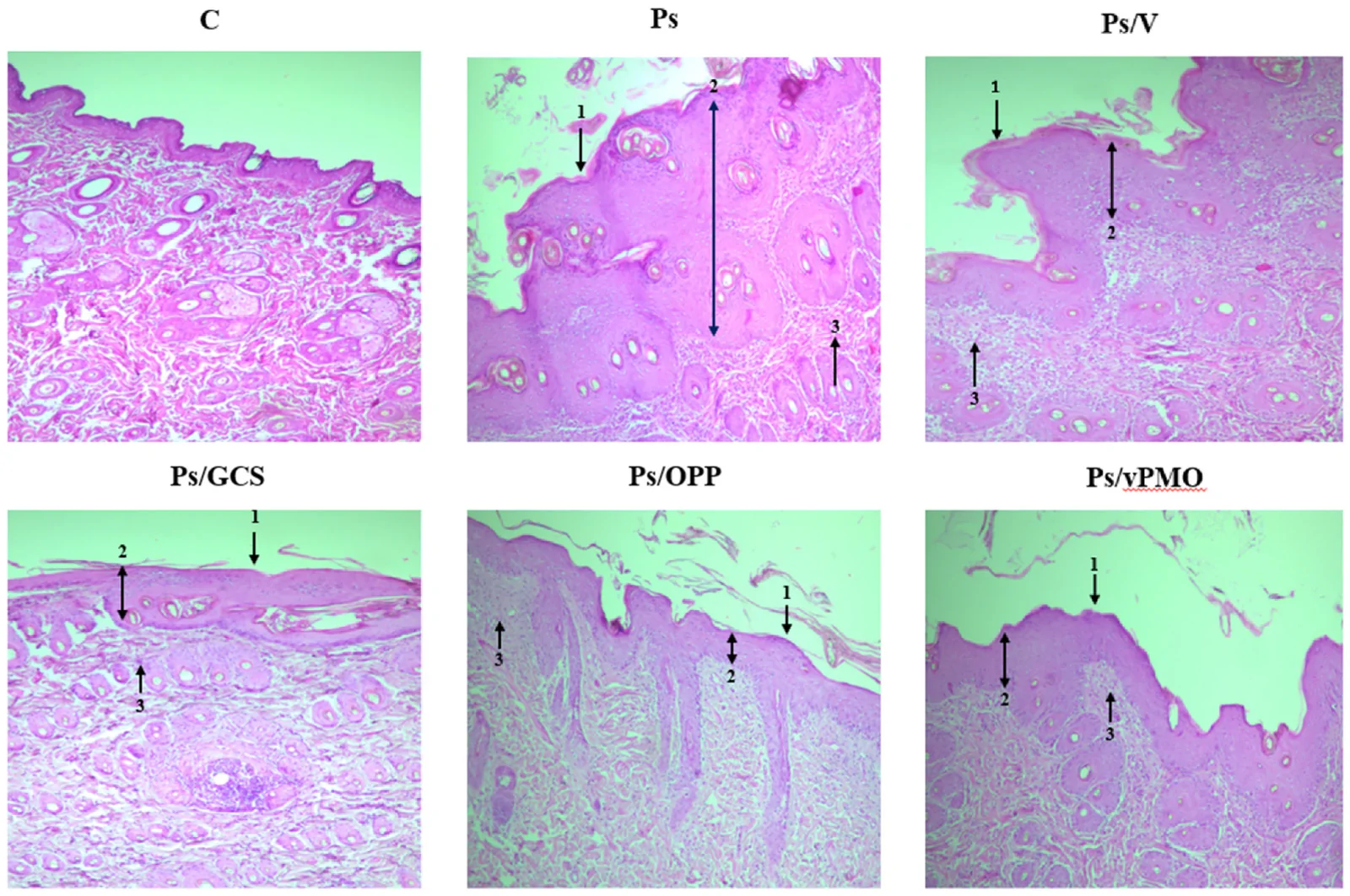
Histopathological view of H&E-stained psoriatic skin lesions of the dorsal portion following imiquimod application and treatment in the respective groups on Day 7. (1) Hyperkeratosis; (2) acanthosis; (3) dermal inflammatory infiltrates.

Formalin-fixed and paraffin-embedded samples were sectioned, stained with hematoxylin and eosin, and microscopically observed. Histological examination revealed morphological features of psoriasiform skin lesions, including hyperkeratosis, acanthosis, and inflammatory infiltration of the dermis, in both the untreated experimental psoriasis group and the treatment groups, complementing the clinical characterization of the experimental model (Figure 4).

### 2.4. Changes in T-Lymphocyte Subpopulations 

**Figure 5:**
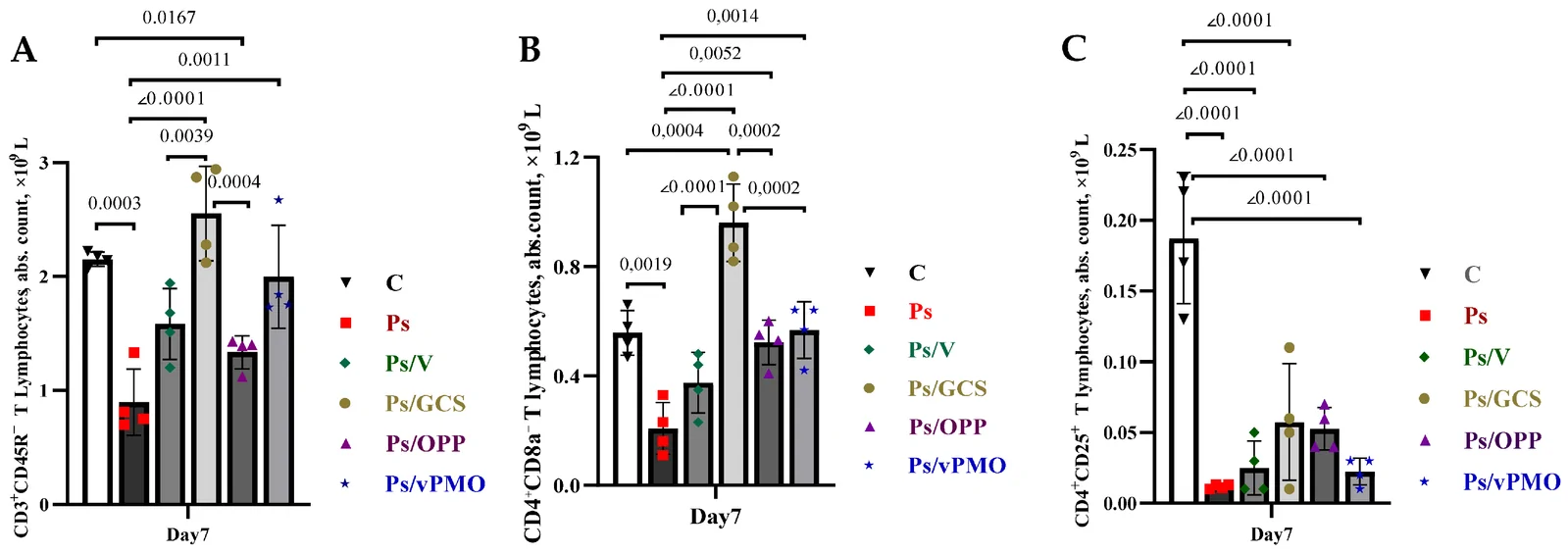
Peripheral blood T-lymphocyte subpopulations on day 7: (**A**) CD3^+^ T lymphocytes; (**B**) CD4^+^CD8^−^ cells; (**C**) CD4^+^CD25^+^ cells. Absolute lymphocyte counts are presented. Data are expressed as mean ± SD. Statistical analysis was performed using one-way ANOVA followed by Tukey’s post hoc test.

Analysis of T-lymphocyte subpopulations revealed intergroup differences in the numbers of CD3^+^CD45R^−^ cells, CD4^+^CD8^−^ cells (T helper cells), and CD4^+^CD25^+^ cells (Figure 5A–C).

In the IMQ-induced psoriasis group, the number of CD3^+^CD45R^−^ cells was significantly reduced by 2.4-fold compared with the control group (0.90 ± 0.029 vs. 2.15 ± 0.06; *p* = 0.0003) (Figure 5A). Treatment with GCS and vPMO resulted in CD3^+^CD45R^−^ cell counts that were 2.8-fold (*p* < 0.0001) and 2.2-fold (*p* = 0.0001) higher, respectively, than those observed in the Ps group. In the OPP-treated group, CD3^+^CD45R^−^ cell counts were approximately twofold lower than those in the GCS-treated group (0.90 ± 0.029 vs. 2.25 ± 0.41; *p* = 0.0004).

The number of CD4^+^CD8^−^ cells in the Ps group was significantly reduced by 2.7-fold compared with the control group (0.21 ± 0.01 vs. 0.56 ± 0.08; *p* = 0.0019) (Figure 5B). In contrast, treatment with GCS, OPP, and vPMO was associated with significantly higher CD4^+^CD8^−^ cell counts compared with the Ps group, with 4.6-fold (0.96 ± 0.14; *p* < 0.0001), 2.5-fold (0.52 ± 0.08; *p* = 0.0052), and 2.7-fold (0.57 ± 0.10; *p* = 0.0014) increases, respectively.

The number of CD4^+^CD25^+^ cells remained significantly lower than that in the control group not only in the Ps group but also in the V-, GCS-, OPP-, and vPMO-treated groups (Figure 5C). Thus, none of the investigated treatments restored CD4^+^CD25^+^ cell counts to the control level.

Overall, the changes in T-lymphocyte subpopulations exhibited a heterogeneous pattern. The most pronounced intergroup differences were observed for CD3^+^CD45R^−^ and CD4^+^CD8^−^ cells, whereas for CD4^+^CD25^+^ cells, significant differences were observed only in comparison with the control group.

### 2.5. Changes in Cytokine Concentrations

**Figure 6:**
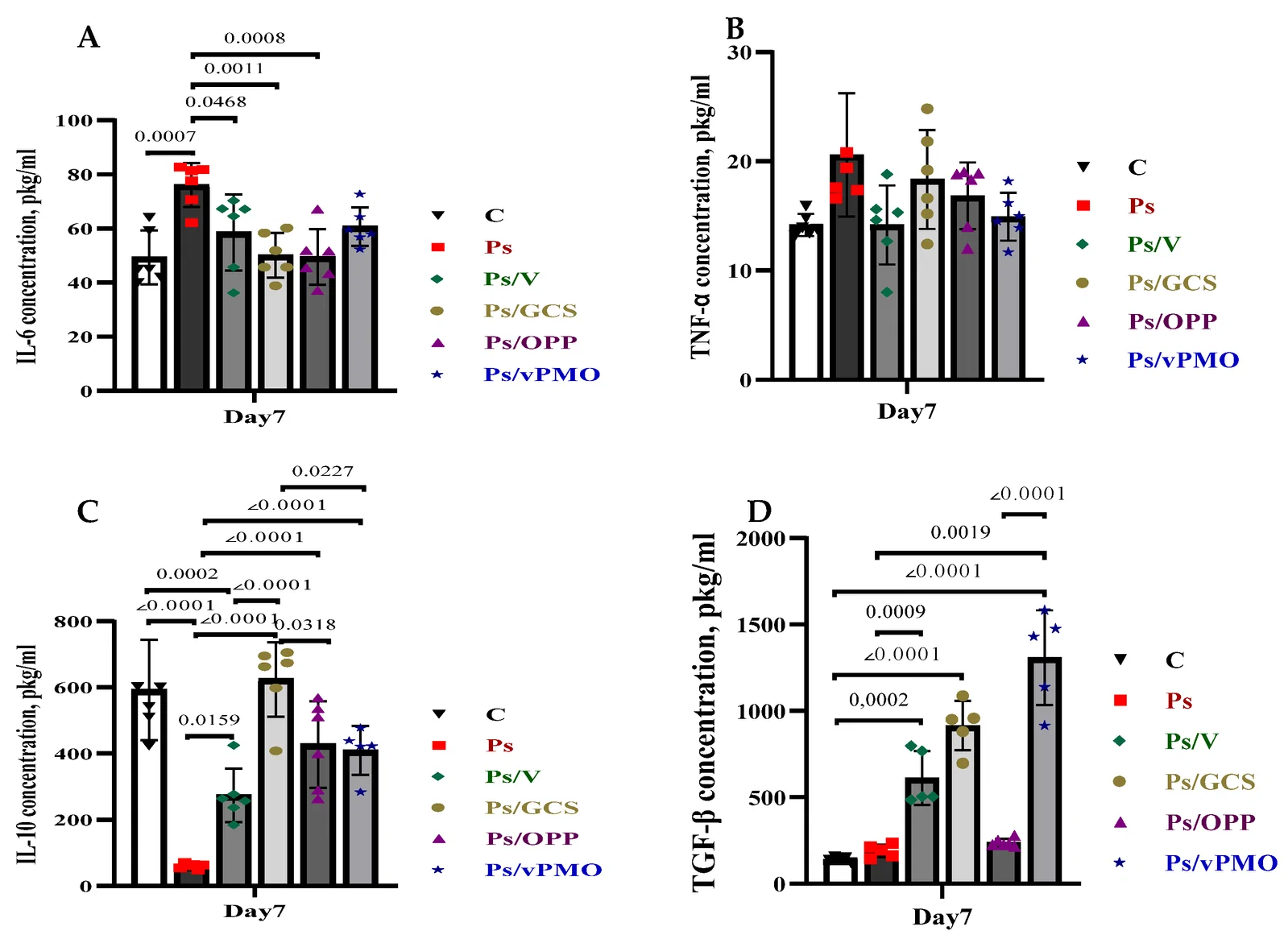
Serum cytokine concentrations on day 7: (**A**) IL-6; (**B**) TNF-α; (**C**) IL-10; (**D**) TGF-β1. Data are expressed as mean ± SD. Statistical analysis was performed using one-way ANOVA followed by Tukey’s post hoc test.

**Figure 7:**
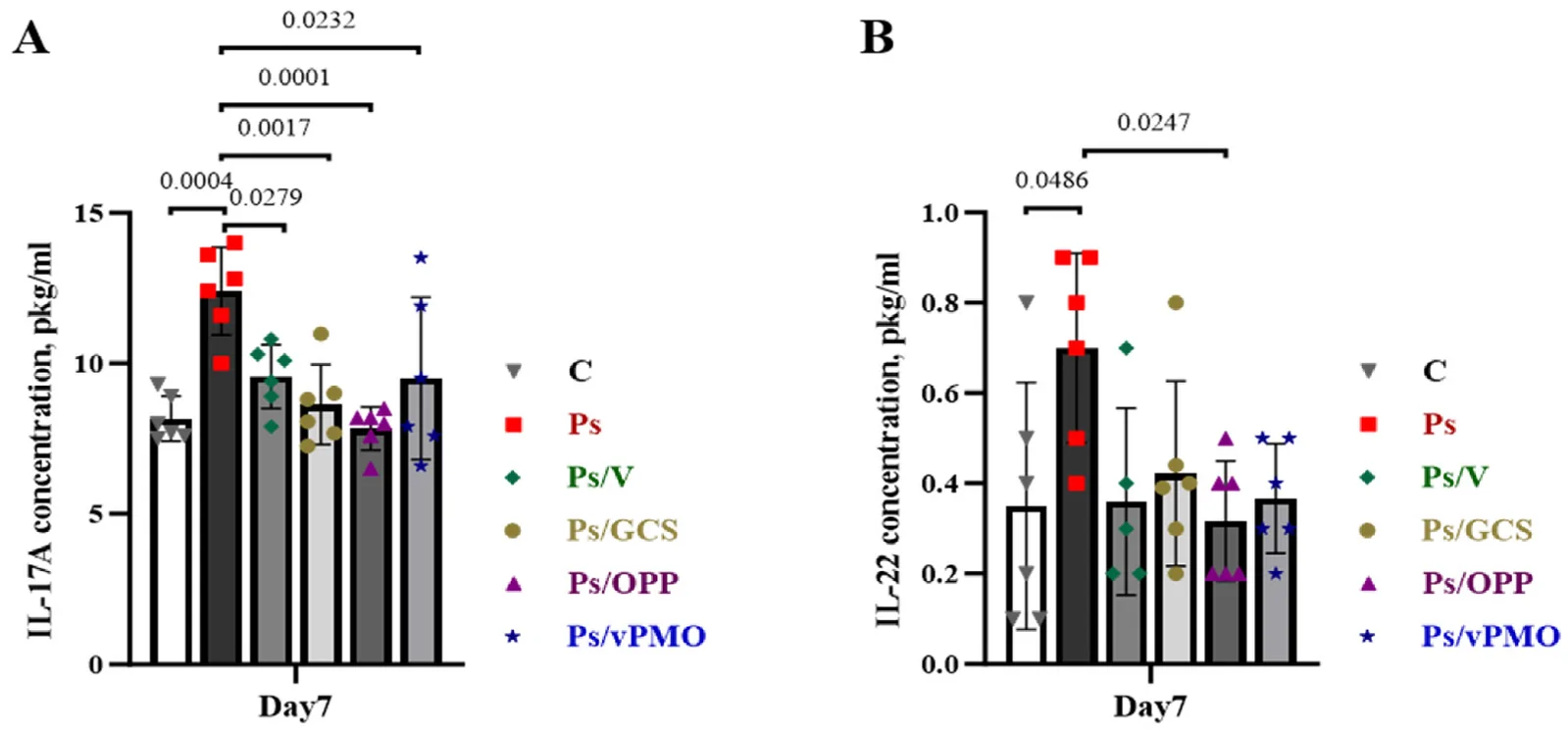
Concentrations of (**A**) IL-17A and (**B**) IL-22 on day 7. Data are expressed as mean ± SD. Statistical analysis was performed using one-way ANOVA followed by Tukey’s post hoc test.

To characterize the systemic inflammatory response, serum concentrations of pro-inflammatory and regulatory cytokines were determined (Figure 6 and Figure 7).

In the Ps group, IL-6 concentrations were elevated compared with the control group. Treatment with V (vehicle), GCS, and OPP reduced IL-6 concentrations by 23.03% (58.52 ± 14.06 vs. 76.03 ± 8.16; *p* = 0.0468), 34.6% (50.13 ± 8.22; *p* = 0.0011), and 35.0% (50.13 ± 8.22; *p* = 0.0008), respectively, compared with the Ps group. In contrast, differences in TNF-α concentrations did not reach statistical significance (Figure 6A,B).

In the Ps group, the IL-10 concentration was approximately 10-fold lower than that in the control group (60.25 ± 7.79 vs. 592.22 ± 151.94; *p* < 0.0001) (Figure 6C). GCS treatment restored the IL-10 concentration to the control level, resulting in a 10.4-fold increase compared with the Ps group (624.07 ± 112.09; *p* < 0.0001). Moreover, the IL-10 concentration in the GCS group was approximately 1.5-fold higher than those in the OPP and vPMO groups (427.82 ± 130.67; *p* = 0.0318 and 409.24 ± 73.52; *p* = 0.0227, respectively). Although V, OPP, and vPMO did not restore IL-10 concentrations to the control level, they resulted in concentrations that were 4.5-fold (273.95 ± 80.75; *p* = 0.0159), 7.1-fold (427.82 ± 130.67; *p* < 0.0001), and 6.8-fold (409.24 ± 73.52; *p* < 0.0001) higher, respectively, than those in the Ps group.

The TGF-β1 concentration did not differ significantly between the Ps and control groups (Figure 6D). Similarly, OPP treatment did not significantly alter TGF-β1 concentrations compared with the Ps group. The most pronounced increase in TGF-β1 was observed following vPMO treatment, with concentrations reaching levels 6.9-fold higher than those in the Ps group (1308.62 ± 274.46 vs. 190.28 ± 36.16; *p* = 0.0019). In contrast to OPP, both GCS and V significantly increased TGF-β1 concentrations, resulting in levels 4.8-fold (914.94 ± 142.11; *p* < 0.0001) and 3.2-fold (611.86 ± 156.59; *p* = 0.0009) higher, respectively, than those in the Ps group.

IL-17A and IL-22 were additionally evaluated as components of the Th17-associated response (Figure 7).

In the Ps group, IL-17A and IL-22 concentrations were 1.5-fold and 2.0-fold higher, respectively, than those in the control group (12.40 ± 1.45 vs. 8.17 ± 0.75; *p* = 0.0004 and 0.70 ± 0.21 vs. 0.35 ± 0.27; *p* = 0.0486, respectively) (Figure 7). OPP treatment reduced IL-17A concentrations by 36.8% (7.83 ± 0.72; *p* = 0.0001) and IL-22 concentrations by 54.7% (0.32 ± 0.13; *p* = 0.0247) compared with the Ps group. Similarly, PMO treatment reduced IL-17A concentrations by 23.4% (9.50 ± 2.70; *p* = 0.0232) and IL-22 concentrations by 47.6% (0.37 ± 0.12; *p* = 0.0247) compared with the Ps group.

Overall, both OPP and PMO treatment were associated with reductions in several pro-inflammatory mediators and increased IL-10 concentrations. The most notable difference between the two compounds was observed for TGF-β1, which showed a particularly pronounced increase following PMO treatment.

## 3. Discussion

The present study combines the chemical characterization of OPP with an initial evaluation of its biological effects. The OPP molecule contains a basic tertiary nitrogen atom, a polar α-hydroxyphosphonate center, and an ether-containing side chain. Such a combination may influence solubility, distribution within the delivery vehicle, transdermal penetration, and interactions with polar regions of biomolecules. However, pKa, logP/logD, solubility, permeability, and the molecular target of OPP were not determined in the present study; therefore, the relationship between its structure and the observed immunological profile should be regarded as a working hypothesis rather than an established mechanism. The chemical functionality of related oxyphosphonates has been demonstrated previously [18,19,20].

IMQ-induced psoriasis was accompanied by a progressive increase in the severity of clinical manifestations, while histological examination revealed hyperkeratosis, acanthosis, and inflammatory infiltration of the dermis. These changes are consistent with the major features of IMQ-induced psoriasiform skin lesions in rats [22]. Concurrently, the absolute numbers of CD3^+^CD45R^−^ and CD4^+^CD8^−^ T cells decreased, IL-6, IL-17A, and IL-22 concentrations increased, and IL-10 concentrations markedly decreased. Alterations in T-cell populations and cytokine regulation are characteristic components of the immune response in psoriasis [4,23,24], while changes in circulating inflammatory cytokines, including IL-17, have also been supported by a meta-analysis of clinical studies [25]. Thus, in the present model, the development of skin lesions was accompanied by alterations in both cellular and humoral components of the immune response.

OPP treatment was associated with clinical improvement accompanied by changes in the profile of the cytokines examined. Reductions in the total PASI score occurred concurrently with decreased IL-6, IL-17A, and IL-22 concentrations and increased IL-10 concentrations. The concordance between the clinical and immunological changes indicates a reduction in the inflammatory response in the Ps/OPP group; however, the changes in IL-6 and IL-10 should be considered in light of the changes in the same direction observed in the Ps/V group. A similar direction of changes was observed with GCS, which reduced the severity of cutaneous manifestations while also affecting cellular and cytokine parameters. However, the earlier and more sustained reduction in the total PASI score observed with GCS distinguished the effect of the reference treatment from that of OPP.

The pattern of changes observed following vPMO treatment was different. The reduction in the total PASI score was accompanied by restoration of CD3^+^CD45R^−^ and CD4^+^CD8^−^ cell counts, increased IL-10 concentrations, and a pronounced increase in TGF-β1, together with reductions in IL-17A and IL-22. In addition, vPMO prevented the body weight loss observed in untreated animals. However, these changes were not accompanied by a significant reduction in individual clinical manifestations. Thus, at the time point examined, the effects of vPMO on immunological parameters were more pronounced than the changes observed directly in the skin.

Differences between OPP and vPMO were particularly evident when cellular and cytokine parameters were compared. Both compounds increased the number of CD4^+^CD8^−^ cells relative to the Ps group, whereas restoration of CD3^+^CD45R^−^ cell counts was characteristic of vPMO treatment. Nevertheless, the number of CD4^+^CD25^+^ cells remained lower than that in the control group with both treatments. Therefore, the increase in IL-10 and other cytokine changes was not accompanied by quantitative restoration of the CD4^+^CD25^+^ cell population. This is important when interpreting the present findings because CD25 expression changes upon T-cell activation, and the phenotypes of activated conventional T cells and regulatory T cells may overlap. Consequently, assessment of CD4^+^CD25^+^ cells without additional markers, including FoxP3 and CD127, does not allow this population to be unequivocally characterized as regulatory T cells [24,26].

The pronounced increase in serum TGF-β1 following vPMO treatment represents one of the most notable differences between the compounds investigated. TGF-β1 contributes to the maintenance of immune homeostasis and regulates the functional state of various immune cell populations, with its biological effects depending on the surrounding cytokine and cellular context [27,28]. In the present study, the increase in TGF-β1 occurred concurrently with increased IL-10 concentrations, decreased levels of pro-inflammatory mediators, and restoration of selected T-cell parameters. Taken together, these changes characterize the immunological profile of the Ps/vPMO group. An increase in TGF-β1 was also observed in the Ps/V group; therefore, this response was not specific to the vPMO formulation. However, the cellular source of the increased TGF-β1 and its functional significance in the observed changes remain to be elucidated.

In the Ps/V group, the decrease in IL-6 concentration was accompanied by increases in IL-10 and TGF-β1 compared with the Ps group, indicating the biological activity of PG/LA in this model. The changes in these cytokines in the same direction across the Ps/V, Ps/OPP, and Ps/vPMO groups indicate that the vehicle contributed to the observed response. Therefore, the changes in these parameters in the Ps/OPP and Ps/vPMO groups cannot be attributed exclusively to the effects of the investigated compounds.

Comparison of the clinical, histopathological, and immunological findings indicates distinct patterns of correction of IMQ-induced alterations by the two investigated compounds. With OPP treatment, reductions in IL-6, IL-17A, and IL-22 and an increase in IL-10 were accompanied by reductions in the cumulative PASI score. With vPMO treatment, reductions in IL-17A and IL-22 and an increase in IL-10 were accompanied by a pronounced increase in TGF-β1 and restoration of CD3^+^CD45R^−^ and CD4^+^CD8^−^ cell counts, whereas the improvement in skin alterations was less pronounced. Thus, in the Ps/OPP group, clinical improvement was accompanied by changes in the examined cytokines, whereas in the Ps/vPMO group, an increase in the numbers of CD3^+^CD45R^−^ and CD4^+^CD8^−^ cells was observed alongside changes in the cytokine profile. The obtained results provide a preliminary characterization of the biological effects of OPP and vPMO in this experimental model. Their interpretation should take into account the biological activity of the PG/LA vehicle, the lack of quantitative data on OPP purity, and the absence of direct experimental validation of the proposed vPMO targets. The observed changes support further investigation of the biological effects of OPP and vPMO, while elucidation of their mechanisms of action will require additional experimental studies.

### Study Limitations

The present findings should be interpreted in light of the seven-day observation period and the scope of the immunological parameters assessed. Furthermore, quantitative data on OPP purity were not provided in the present study, and the proposed molecular targets of vPMO were not directly validated experimentally. These aspects limit the interpretation of the mechanisms of the observed effects. The observed changes in T-cell subpopulations and cytokine profiles reflect systemic alterations in the immune response and provide a basis for its more comprehensive characterization in future studies, including the assessment of additional phenotypic and functional markers, as well as local immune and inflammatory changes in the skin. Ongoing experimental studies include transcriptomic analysis aimed at further characterizing the molecular changes associated with the administration of the investigated compounds. The differences observed between OPP and vPMO also warrant further investigation of the physicochemical and pharmacokinetic properties of OPP, as well as clarification of the putative molecular target of vPMO. Collectively, these findings provide a foundation for further studies aimed at elucidating the mechanisms of action and advancing the preclinical characterization of the investigated compounds.

## 4. Materials and Methods

### 4.1. Synthesis of Dimethyl [4-Hydroxy-1-(3-isopropoxypropyl)piperidin-4-yl]phosphonate (OPP)

The progress of the reaction was monitored by thin-layer chromatography on Al_2_O_3_ using acetone as the eluent system, spots were visualized by UV and iodine vapor. The melting point was determined using a KRUSS M3000 melting point meter (A. KRÜSS Optronic GmbH, Hamburg, Germany). The IR spectrum was recorded as a KBr pellet on a Nicolet 5700 FT-IR spectrometer (Thermo Electron Corporation, Madison, WI, USA). Elemental analysis was performed using a FlashSmart Elemental Analyzer (Thermo Fisher Scientific Inc., Waltham, MA, USA). ^1^H, ^13^C, COSY, HMQC, and HMBC NMR spectra were recorded on a JNM-ECA JEOL 400 spectrometer (JEOL Ltd., Tokyo, Japan) at 399.78 MHz for ^1^H and 100.53 MHz for ^13^C in CDCl_3_. The ^31^P NMR spectrum was recorded on a Bruker Avance 400 high-resolution NMR spectrometer (Bruker, Germany) operating at 161.90 MHz in CDCl_3_.

A three-necked flask equipped with a mechanical stirrer, reflux condenser, dropping funnel, and thermometer was charged with 1-(3-isopropoxypropyl)piperidin-4-one (19.9 g, 0.100 mol) [21], dimethyl phosphite (16.5 g, 0.150 mol, 1.5 equiv.), and hexane (88 mL). The mixture was cooled to 10 °C, after which a saturated solution of sodium methoxide (approximately 3.5 mL; 1.1 g, 0.019 mol) was added slowly at such a rate that the temperature did not exceed 30 °C. After completion of the addition, the reaction mixture was stirred at room temperature for 4 h. Reaction completion was monitored by TLC based on the disappearance of the spot corresponding to the starting ketone. The reaction mixture was kept at −23 °C for 12 h to induce crystallization of the product. The precipitate was collected by filtration, washed with cold hexane, dried in a vacuum desiccator, and recrystallized from hexane. OPP was obtained in a yield of 21.7 g (70%) as white crystals; mp 72–73 °C; Rf 0.36.

FTIR (KBr, ν, cm^−1^): 3319 (O–H), 2966, 2878, 2852, 2812, 2773 (C–H), 1222 (P=O), 1179 (P–OCH_3_), 1079, 1039 (C–O–C).

^1^H NMR (399.78 MHz, CDCl_3_, δ, ppm): 1.00–1.07 (m, 6H, H-12, H-17), 1.60–1.65 (m, 2H, H-8), 1.77–1.97 (m, 4H, H-3, H-5), 2.23–2.33 (m, 4H, H-7, H-2_ax_, H-6_ax_), 2.54–2.58 (m, 2H, H-2_eq_, H-6_eq_), 3.03–3.09 (br m, 1H, OH), 3.29–3.40 (m, 3H, H-9, H-11), 3.51–3.74 (m, 6H, P(OCH_3_)_2_).

^13^C NMR (100.53 MHz, CDCl_3_, δ, ppm): 22.11 (C-12, C-17), 27.58 (C-8), 31.52 (C-3, C-5), 47.52 (C-2, C-6), 53.59 (P(OCH_3_)_2_), 55.64 (C-7), 66.47 (C-9), 69.17 (d, ^1^J(C,P) = 168.9 Hz, C-4; components at 68.33 and 70.01 ppm), 71.28 (C-11).

^31^P NMR (161.90 MHz, CDCl_3_, δ, ppm): 27.91 (P).

Elemental analysis for C_13_H_28_NO_5_P, calculated (%): C, 50.48; H, 9.12; N, 4.53; P, 10.01. Found (%): C, 50.59; H, 8.91; N, 4.32; P, 9.91.

### 4.2. Animals and Ethical Aspects

Male Rattus norvegicus rats aged 8–12 weeks and weighing 180–220 g were housed in plastic cages at the B.A. Atchabarov Research Institute of Fundamental and Applied Medicine, Asfendiyarov Kazakh National Medical University, Almaty, Kazakhstan. Animals were acclimated at least 14 days before the experiments. Animals were housed at 22.5–23.0 °C, 50–70% relative humidity, and a 12 h light/dark cycle, with ad libitum access to standard feed and water.

The procedures complied with the Rules for Conducting Biomedical Experiments and the requirements for preclinical and clinical laboratories approved by Order No. 142 of the Minister of Health of the Republic of Kazakhstan dated 2 April 2018 [29]. The protocol was approved by the Local Bioethics Committee of Asfendiyarov Kazakh National Medical University (Protocol No. 36 (172), dated 28 November 2025).

### 4.3. Compounds Investigated

Dimethyl [4-hydroxy-1-(3-isopropoxypropyl)piperidin-4-yl]phosphonate (OPP) (C_13_H_28_NO_5_P; M_r_ = 309.34) was synthesized at the Laboratory of Synthetic and Natural Medicinal Compounds Chemistry, A.B. Bekturov Institute of Chemical Sciences (Almaty, Kazakhstan), according to the procedure described in Section 4.1. For the biological experiment, a sample of OPP was used [synthesized on 9 April 2025; mp 72–73 °C; TLC (Al_2_O_3_), Rf 0.36 (acetone)]. OPP was administered topically at a dose of 50 mg/kg. The OPP dose of 50 mg/kg was selected for this exploratory study based on previous data obtained with structurally related piperidine derivatives, including the EPPPβCD complex [13]. Because OPP contains an α-hydroxyphosphonate moiety and is neither EPPP nor its β-cyclodextrin complex, this rationale is empirical and does not imply pharmacological equivalence between these compounds.

vPMO was synthesized by Gene Tools, LLC (Philomath, OR, USA). Following synthesis, the oligo was filtered, selectively precipitated, resuspended in pure water, and lyophilized. No acids or salts were used during the purification process. The molecular mass of the synthesized vPMO was confirmed by mass spectrometry (Figures S8 and S9). The antisense 25-mer vPMO targeting exon 10 of the rat *IL1RL2* was designated EX10AO2 and had the following sequence: 5′-AAGATCTTCAACACCACTGTGTCCA-3′.

### 4.4. Experimental Design

The animals were randomly allocated into six groups (n = 6 per group): a control group (C), experimental psoriasis group (Ps), and four treatment groups receiving the vehicle (Ps/V), glucocorticosteroid (Ps/GCS), OPP (Ps/OPP), vPMO (Ps/vPMO), respectively, in the presence of induced psoriasis. Control rats received Vaseline application on the skin.

In the Ps/GCS group, 0.05% Dermovate^®^ cream (Clobetasol propionate, GSK, registration number RK-LS-5 N°009575, Poland; Delpharm Poznan Spolka Akcyjna, Poznan, Poland) was applied topically to the dorsal skin approximately 1 h after IMQ application, once daily for 7 consecutive days. In the Ps/V group, 0.1 mL of the vehicle, consisting of propylene glycol and linoleic acid (PG/LA, 95:5), was applied to the dorsal skin approximately 1 h after IMQ application, once daily for 7 consecutive days. The same vehicle formulation was used for the administration of OPP and vPMO. The PG/LA vehicle (95:5) was selected based on previous evidence supporting its use for topical delivery of vPMOs [30].

All procedures were performed under general anesthesia using Zoletil (tiletamine/zolazepam, 20–40 mg/kg, intramuscularly) in combination with xylazine (5–10 mg/kg, intramuscularly); atropine (0.5–1 mg/kg, intramuscularly) was used for premedication [31]. After the procedures, the animals were returned to their cages and monitored until full recovery from anesthesia.

Clinical manifestations were assessed on Days 3, 5 and 7 using the modified PASI scale [32], while body weight was measured simultaneously. On day 7, the animals were euthanized under general anesthesia, and blood samples were collected. T-cell subpopulations and cytokine concentrations were determined at the Life Science Laboratory of the B.A. Atchabarov Institute of Fundamental and Applied Medicine.

### 4.5. Induction of Psoriasis-like Dermatitis

One day before the start of topical IMQ application, the central area of the rats’ dorsal skin was shaved and depilated using a depilatory cream over an area of approximately 4 cm^2^. To induce psoriasis-like dermatitis, 5% imiquimod (Aldara; Viatris, Mérignac, France) was applied daily to the shaved area at a dose of 62.25 mg/day for 7 days [33,34]. A control group of rats did not receive IMQ.

### 4.6. Topical Delivery of vPMO and OPP

Lyophilized vPMO was dissolved in sterile water for injection to prepare a stock solution at a concentration of 0.5 mM. The required volume of the vPMO stock solution was calculated individually according to the body weight of each animal to achieve a dose of 0.03 mg/kg and was added to 0.1 mL of the vehicle (95%propylene glycol + 5% linoleic acid). OPP was dissolved directly in the same vehicle using the same component ratio. For each animal, 0.1 mL of the vehicle was used, to which the calculated amount of OPP was added to achieve a dose of 50 mg/kg. The resulting mixtures were applied to the prepared skin area 1 h after IMQ application, once daily for 7 consecutive days. An applicator was used to minimize the risk of the animals licking off the preparation and to ensure reproducibility of its application.

### 4.7. Flow Cytometry

Immunophenotyping was performed using fresh peripheral blood within 1–2 h after collection. Red blood cells were lysed using High-Yield Lyse reagent, and the cells were washed twice with 0.9% NaCl and resuspended in chilled 0.9% NaCl.

Surface staining was performed using monoclonal antibodies against CD3, CD45R (B220), CD4, CD8a, and CD25 (Table 1). Samples were incubated with antibodies for 30 min at +4 °C in the dark, washed, and analyzed using an Attune™ NxT flow cytometer (Thermo Fisher Scientific) with Attune™ NxT Cytometric Software 6.0.1. Fluorescence-minus-one (FMO) controls were used to establish the boundaries of positive populations.

The lymphocyte population was identified based on FSC-A/SSC-A, and singlets were identified using FSC-A/FSC-H. T lymphocytes were defined as CD3^+^CD45R^−^ cells; CD45R^+^ cells were excluded. CD4^+^CD8a^−^ cells within the CD3^+^CD45R^−^ gate were considered T helper cells. CD25 expression was assessed within the CD4^+^ gate; the CD25^+^/CD25^−^ boundary was established using an FMO control in the PE-A channel. The proportion of each subpopulation was expressed relative to the corresponding parent gate.

Throughout this manuscript, CD3^+^CD45R^−^ cells are designated as CD3^+^ T lymphocytes, CD4^+^CD8a^−^ cells as CD4^+^CD8^−^ cells (T helper cells), and CD4^+^CD25^+^ cells as T cells with regulatory potential.

#### Flow Cytometry Gating Strategy

Flow cytometry data analysis was performed using a sequential gating strategy. First, the lymphocyte population was identified from all cells acquired based on characteristic forward- and side scatter parameters (FSC-A and SSC-A). Next, single cells (singlets) were subsequently selected from the lymphocyte gate using the ratio of FSC-A to FSC-H parameters to exclude cell aggregates and doublets.

Within the singlet population, T- and B-lymphocytes were distinguished based on CD3 and CD45R expression, respectively. CD3+ cells were selected for subsequent analysis of T-cell subpopulations. Within the CD3+ T-cell population, CD4+CD25+ T cells and CD8a+ T lymphocytes were identified based on the expression of the corresponding surface markers. The CD8a+ T-cell population was further analyzed for CD25 expression to assess the proportion of CD25-expressing cells within this subpopulation (Figure S10).

The same gating strategy and criteria were consistently applied to all samples, ensuring the comparability of results across experimental groups.

### 4.8. Cytokine Determination by ELISA

Serum concentrations of IL-6, TNF-α, TGF-β1, IL-10, IL-17A, and IL-22 were determined using sandwich ELISA with commercially available kits from Thermo Fisher Scientific/Invitrogen (Table 2). The assays were performed according to the manufacturers’ instructions.

After binding of the analyte to immobilized antibodies, biotinylated detection antibodies and streptavidin-HRP were sequentially added. Color development was performed using TMB and measured at 450 nm. Concentrations were calculated using individual standard curves.

For TGF-β1 determination, samples underwent preliminary acid activation followed by neutralization according to the protocol for kit BMS623-3. Standard ranges were 31.3–2000 pg/mL for IL-6, 39–2500 pg/mL for TNF-α, and 15.6–1000 pg/mL for IL-10.

### 4.9. Statistical Analysis

Each experimental group consisted of six animals. For between-group comparisons at a single time point, one-way ANOVA with Tukey’s post hoc test was used. For parameters measured repeatedly over time, including clinical parameters and body weight, two-way ANOVA was applied to assess the effects of group, time, and their interaction, followed by Tukey’s post hoc test. Differences were considered statistically significant at *p* ≤ 0.05.

Statistical analyses and visualization were performed using GraphPad Prism 10.6.1. Data are presented as mean ± standard deviation (M ± SD).

## 5. Conclusions

Dimethyl [4-hydroxy-1-(3-isopropoxypropyl)piperidin-4-yl]phosphonate (OPP) was synthesized by hydrophosphorylation of the corresponding piperidin-4-one and isolated in 70% yield. The IR, 1H, 13C, COSY, HMQC, and HMBC spectroscopic data, together with the elemental analysis results, were mutually consistent with the proposed oxyphosphonate structure.

In the IMQ-induced psoriasiform skin lesion model, administration of OPP and vPMO was associated with a reduction in the overall severity of clinical manifestations and alterations in the cellular and cytokine components of the immune response. The observed clinical and immunological changes suggest the potential therapeutic value of OPP and vPMO and support their further preclinical investigation.

When interpreting the obtained results, the biological activity of the vehicle should be taken into account. Further elucidation of the mechanisms of action of OPP and vPMO requires additional studies, including direct experimental validation of the proposed molecular targets of vPMO and quantitative assessment of OPP purity.

## Figures and Tables

**Table 1:** Antibodies and fluorochromes used for immunophenotyping.

Catalog No.	Antibody	Fluorochrome	Manufacturer	Clone
17-0030-82	CD3	APC	eBioscience™	eBioG4.18 (G4.18)
25-0460-82	CD45R (B220)	PE-Cyanine7	eBioscience™	HIS24
11-0040-82	CD4	FITC	eBioscience™	OX35
46-0084-82	CD8a	PerCP-eFluor™ 710	eBioscience™	OX8
12-0390-82	CD25	PE	eBioscience™	OX39

**Table 2:** ELISA kits used in this study.

Cytokine	Kit	Sample	Manufacturer	Catalog No.
IL-6	Rat IL-6 ELISA Kit	Serum	Invitrogen (Thermo Fisher Scientific, Frederick, MD, USA)	BMS625
TNF-α	Rat TNF alpha ELISA Kit	Serum	Invitrogen (Thermo Fisher Scientific, Frederick, MD, USA)	BMS622
TGF-β1	Rat TGF beta 1 ELISA Kit	Serum	Invitrogen (Thermo Fisher Scientific, Frederick, MD, USA)	BMS623-3
IL-10	Rat IL-10 ELISA Kit	Serum	Invitrogen (Thermo Fisher Scientific, Frederick, MD, USA)	BMS629
IL-17A	Rat IL-17A ELISA Kit	Serum	Invitrogen (Thermo Fisher Scientific, Frederick, MD, USA)	BMS635
IL-22	Rat IL-22 ELISA Kit	Serum	Invitrogen (Thermo Fisher Scientific, Frederick, MD, USA)	ERA27RB

## Data Availability

Spectroscopic data related to the compounds investigated in this study are available in the Supplementary Materials. The biological and experimental data supporting the findings of this study are available from the corresponding author upon reasonable request. The original study materials and research data are retained by the authors and may be used in subsequent research.

## Supplementary Materials

The following supporting information can be downloaded at: https://www.mdpi.com/article/10.3390/molecules31213926/s1, Figure S1: IR (KBr, ν, cm−1) spectrum of dimethyl [4-hydroxy-1-(3-isopropoxypropyl)piperidin-4-yl]phosphonate (OPP); Figure S2: ^1^H NMR (399.78 MHz, CDCl_3_) spectrum of dimethyl [4-hydroxy-1-(3-isopropoxypropyl)piperidin-4-yl]phosphonate (OPP); Figure S3: ^13^C NMR (100.53 MHz, CDCl_3_) spectrum of dimethyl [4-hydroxy-1-(3-isopropoxypropyl)piperidin-4-yl]phosphonate (OPP); Figure S4: ^1^H-^1^H COSY spectrum of dimethyl [4-hydroxy-1-(3-isopropoxypropyl)piperidin-4-yl]phosphonate (OPP); Figure S5: ^1^H-^13^C HMQC spectrum of dimethyl [4-hydroxy-1-(3-isopropoxypropyl)piperidin-4-yl]phosphonate (OPP); Figure S6: ^1^H-^13^C HMBC spectrum of dimethyl [4-hydroxy-1-(3-isopropoxypropyl)piperidin-4-yl]phosphonate (OPP); Figure S7. ^31^P NMR (161.90 MHz, CDCl_3_) spectrum of dimethyl[4-hydroxy-1-(3-isopropoxypropyl)piperidin-4-yl]phosphonate (OPP); Figure S8. Mass spectrometric characterization of Vivo-Morpholino (vPMO); Figure S9. Oligonucleotides’ properties; Figure S10. Flow cytometry gating strategy.

## Abbreviations

The following abbreviations are used in this manuscript:

CD	Cluster of Differentiation
IL	Interleukin
IL1RL2	Interleukin 1 receptor like 2
IMQ	Imiquimod
IRF4	Interferon regulatory factor 4
OPP	Piperidine α-hydroxyphosphonate
PASI	Psoriasis Area and Severity Index
PG/LA	Propylene glycol/linoleic acid
TGF-β1	Transforming growth factor beta 1
TNF-α	Tumor necrosis factor alpha
vPMO	Vivo-phosphorodiamidate morpholino oligomer
