# Supplementary material for: Comparative Evaluation of the Early Immunological Effects of a Novel Piperidine α-Hydroxyphosphonate and a Phosphorodiamidate Morpholino Oligomer in an Imiquimod-Induced Psoriasis Model
Source: Molecules. 2026 Nov 1;31(21):3926. doi: 10.3390/molecules31213926 (PMC13765465; doi:10.3390/molecules31213926)
Supplement: Supplementary file 1 [file molecules-31-03926-s001.zip › molecules-4644147-supplementary.pdf]

Supporting information

The IR spectrum was recorded as a KBr pellet on a Nicolet 5700 FT-IR spectrometer, operating range 400-4000 cm^{-1}.

The ^{1}H and ^{13}C NMR spectra of the samples were recorded using a JNM-ECA 400 (Jeol) spectrometer, operating at frequencies of 399.78 MHz for ^{1}H and 100.53 MHz for ^{13}C, in deuterated chloroform ($CDCl_3$).

The ^{31}P NMR spectrum was recorded on a Bruker Avance 400 high-resolution NMR spectrometer, operating at 161.90 MHz in $CDCl_3$.

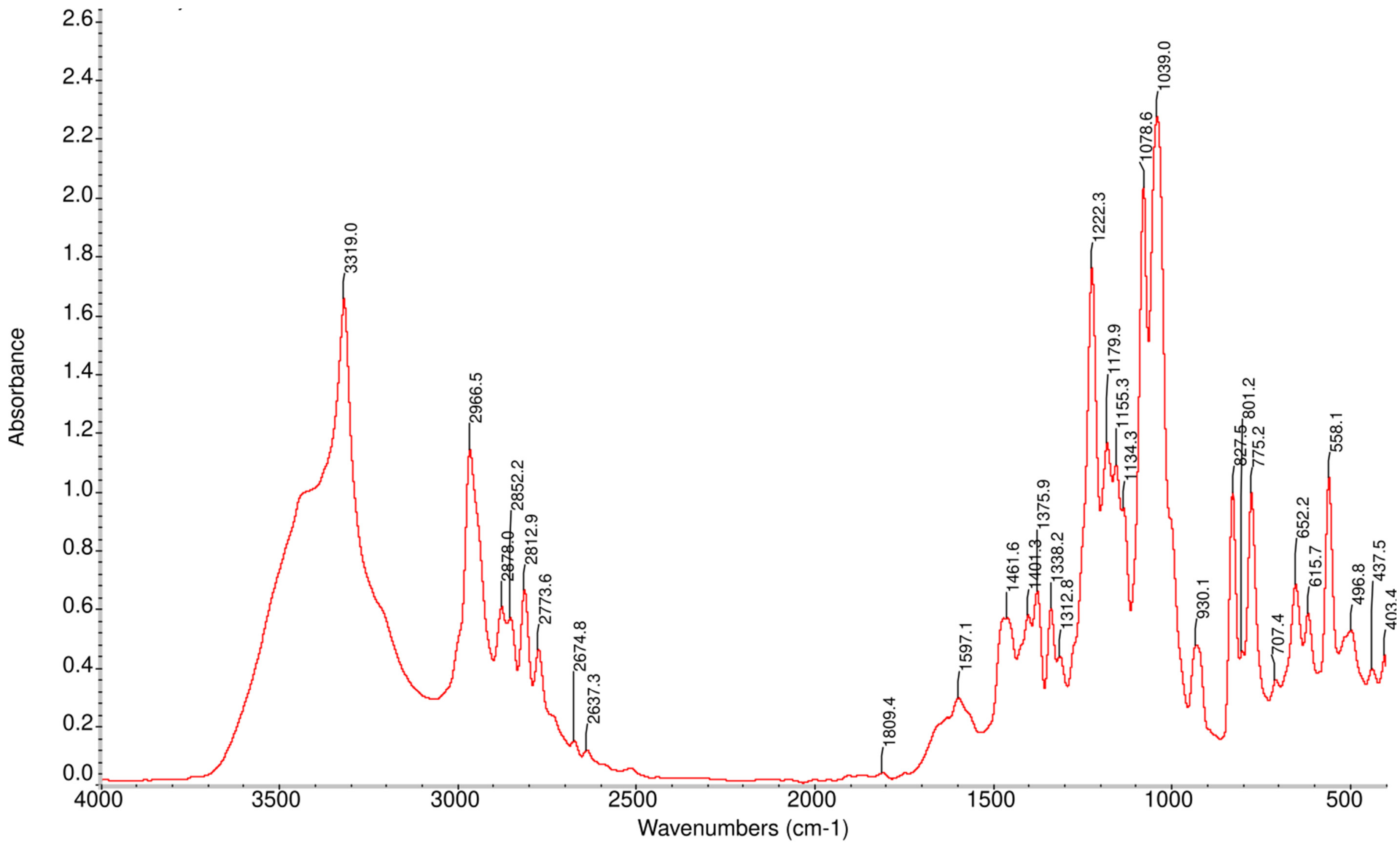

Figure S1. IR (KBr, ν, cm-1) spectrum of dimethyl[4-hydroxy-1-(3-isopropoxypropyl)piperidin-4-yl]phosphonate (OPP).

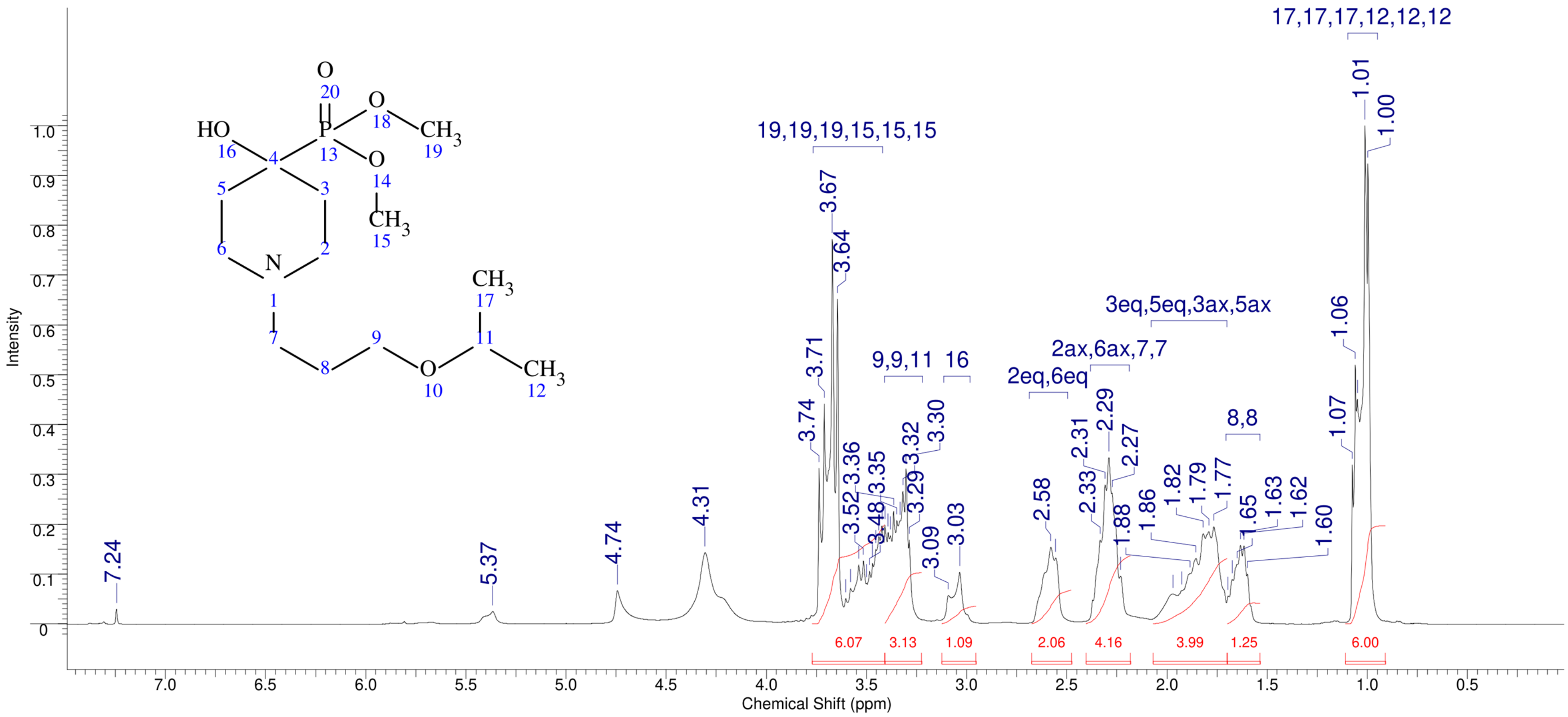

Figure S2. ^{1}H NMR (399.78 MHz, $CDCl_3$) spectrum of dimethyl[4-hydroxy-1-(3-isopropoxypropyl)piperidin-4-yl]phosphonate (OPP).

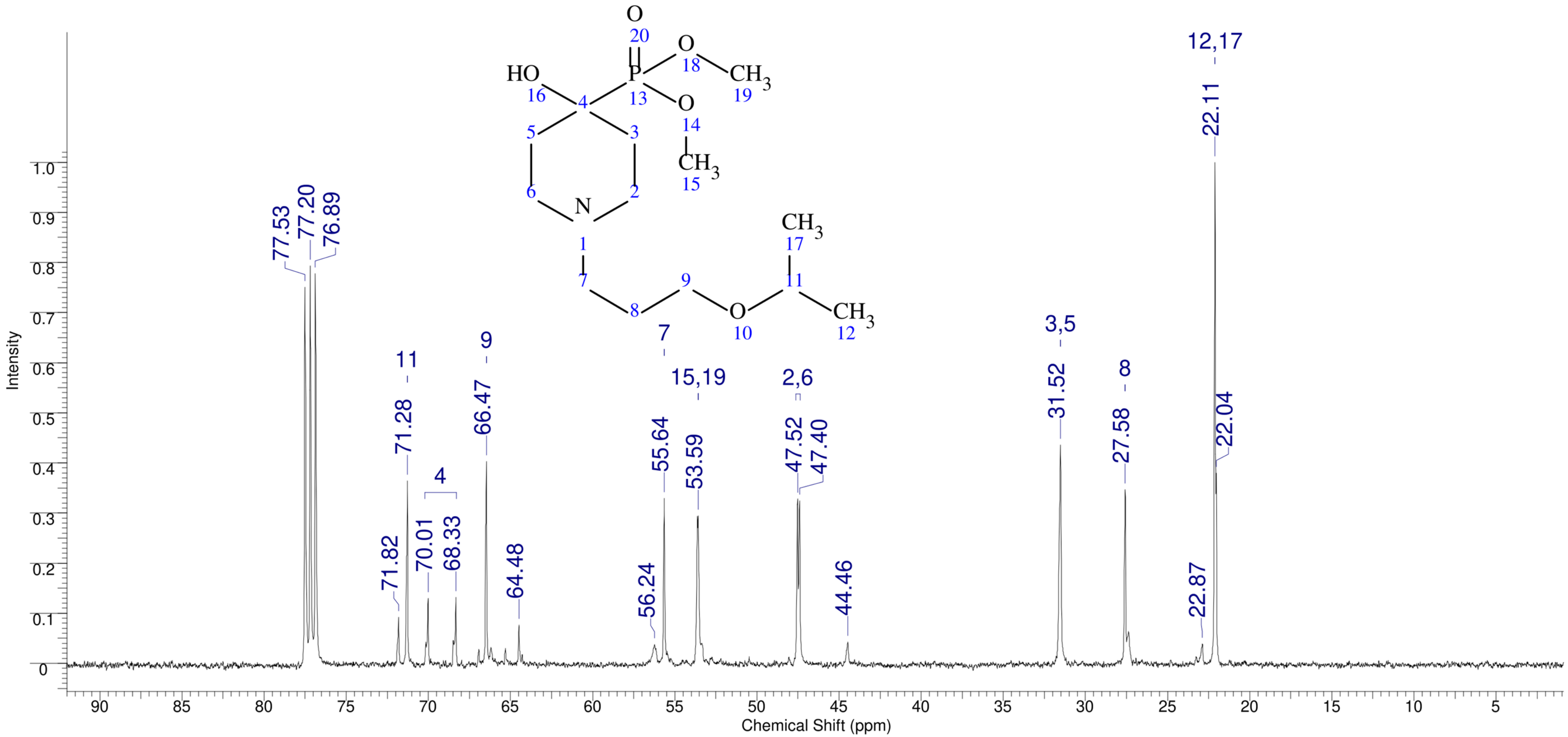

Figure S3. ^{13}C NMR (100.53 MHz, $CDCl_3$) spectrum of dimethyl[4-hydroxy-1-(3-isopropoxypropyl)piperidin-4-yl]phosphonate (OPP).

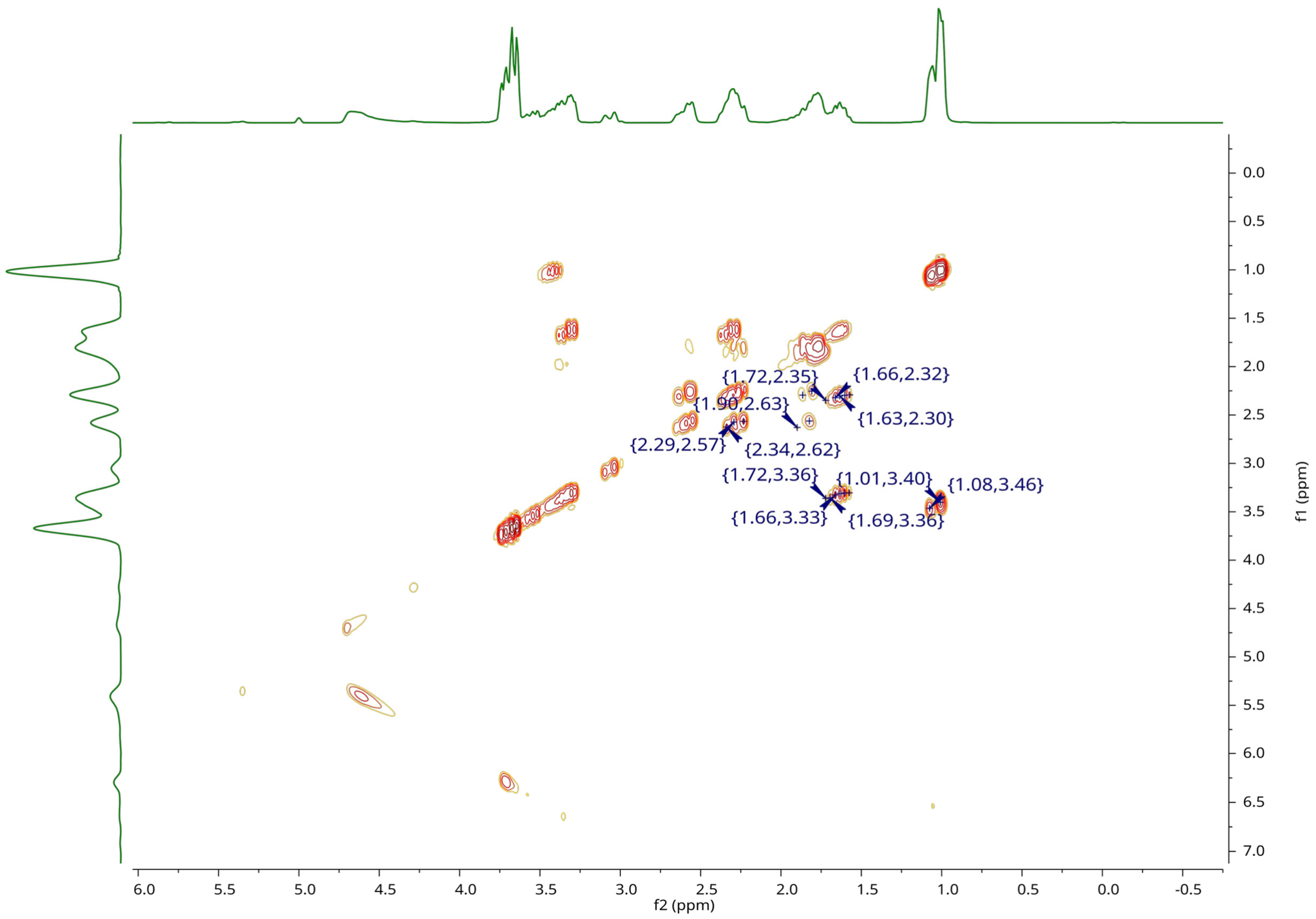

Figure S4. ^{1}H-^{1}H COSY spectrum of dimethyl[4-hydroxy-1-(3-isopropoxypropyl)piperidin-4-yl]phosphonate (OPP).

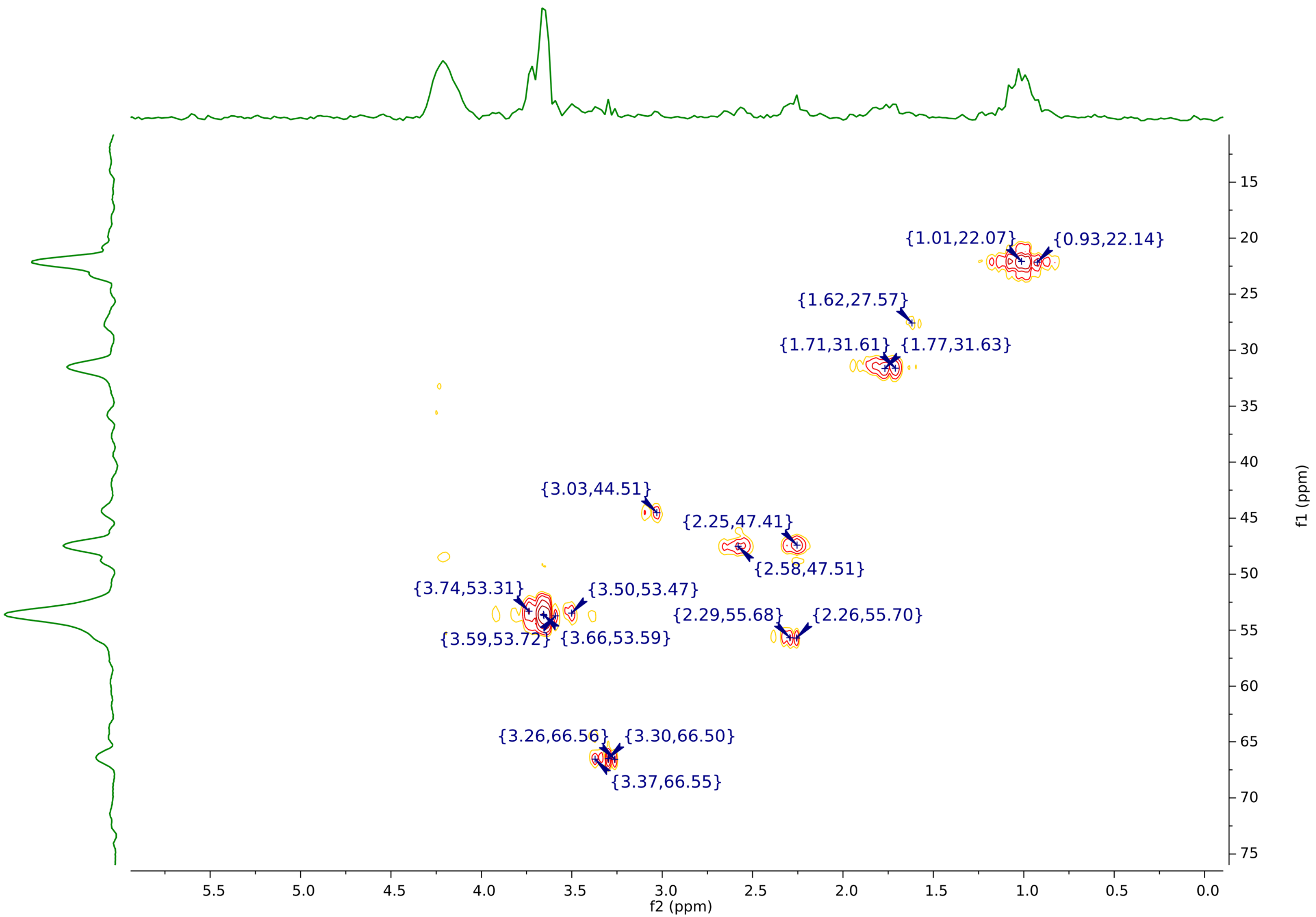

Figure S5. ^{1}H-^{13}C HMQC spectrum of dimethyl[4-hydroxy-1-(3-isopropoxypropyl)piperidin-4-yl]phosphonate (OPP).

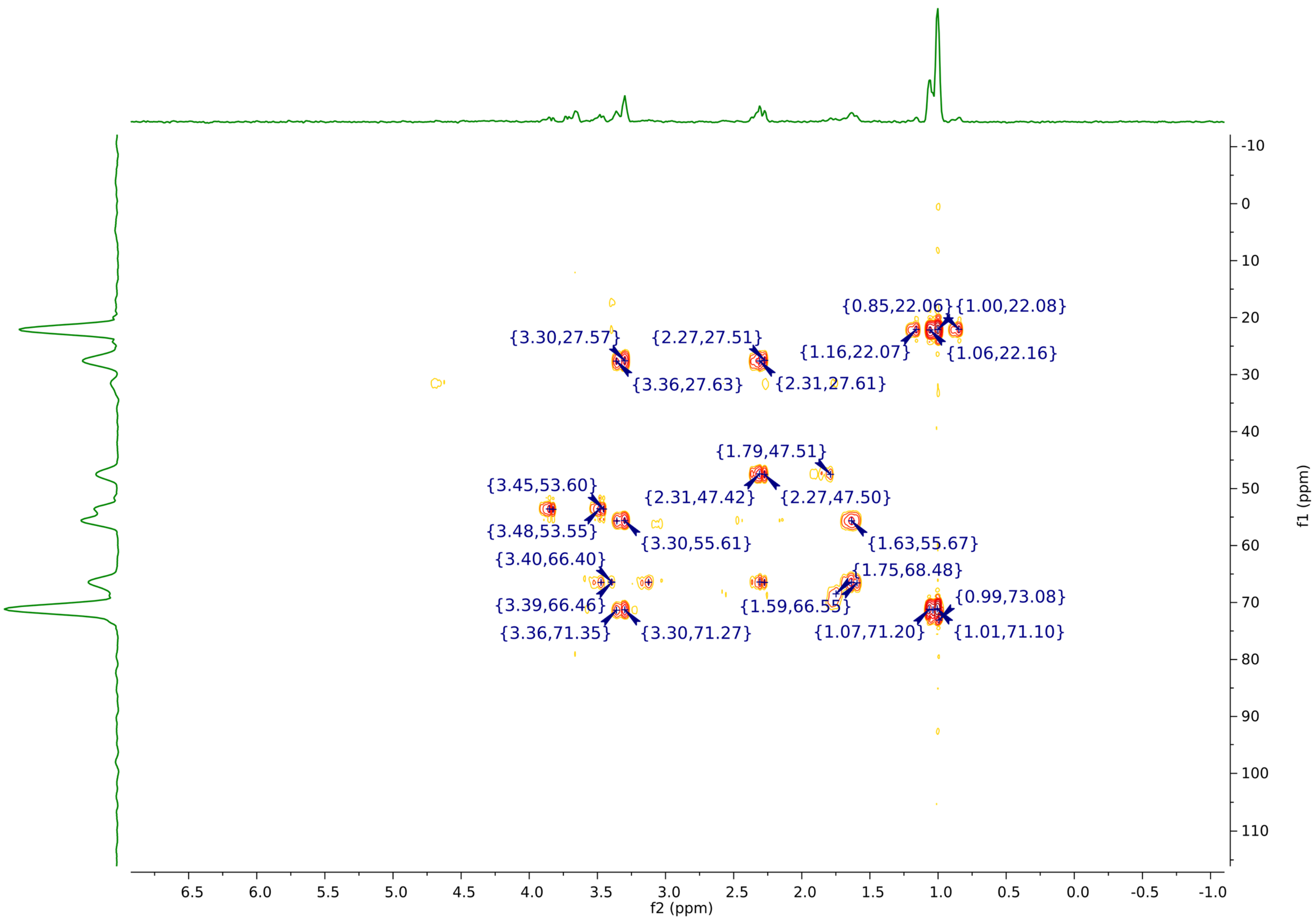

Figure S6. ^{1}H-^{13}C HMBC spectrum of dimethyl[4-hydroxy-1-(3-isopropoxypropyl)piperidin-4-yl]phosphonate (OPP).

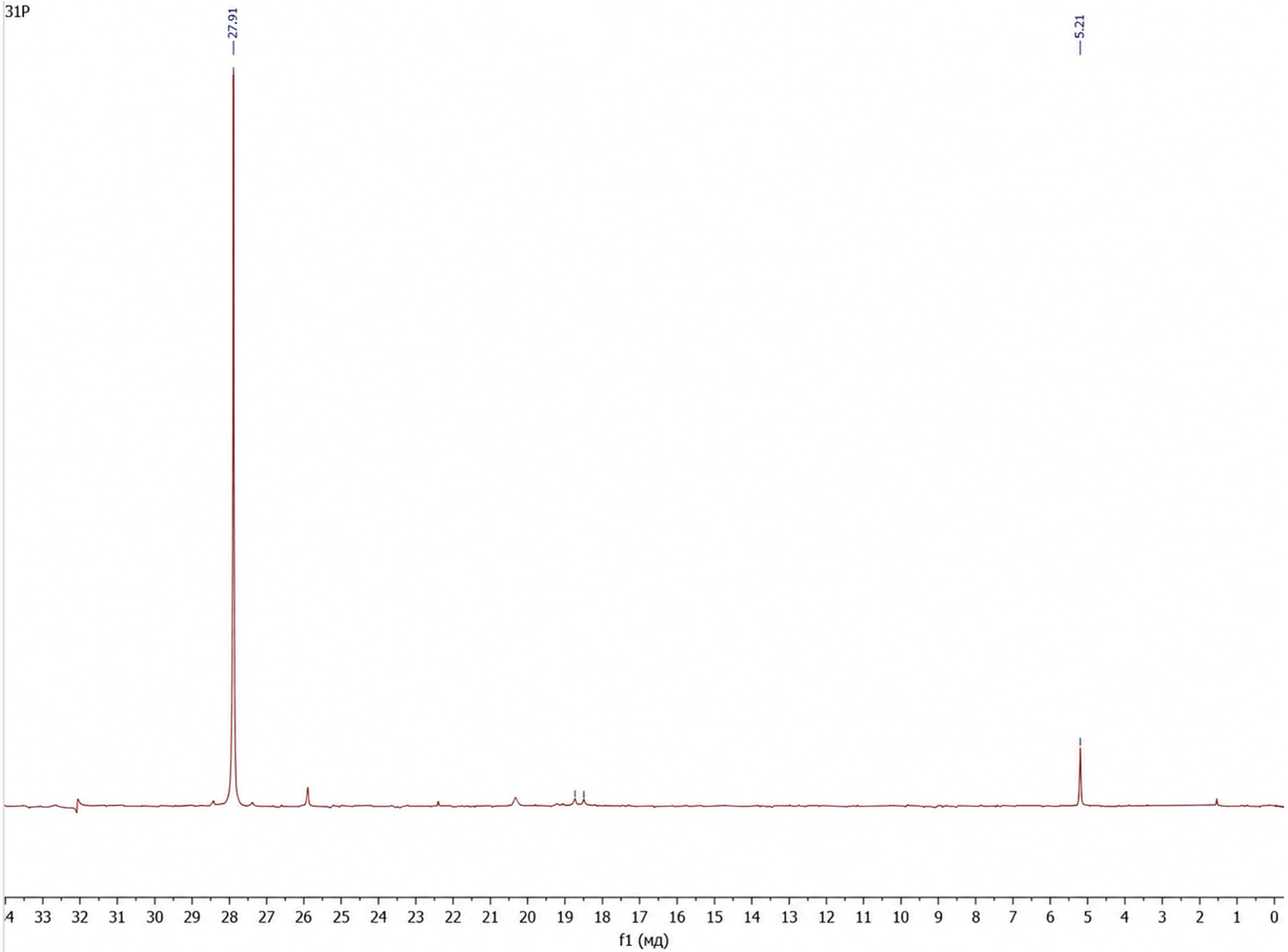

Figure S7. ^{31}P NMR (161.90 MHz, $CDCl_3$) spectrum of dimethyl[4-hydroxy-1-(3-isopropoxypropyl)piperidin-4-yl]phosphonate (OPP).

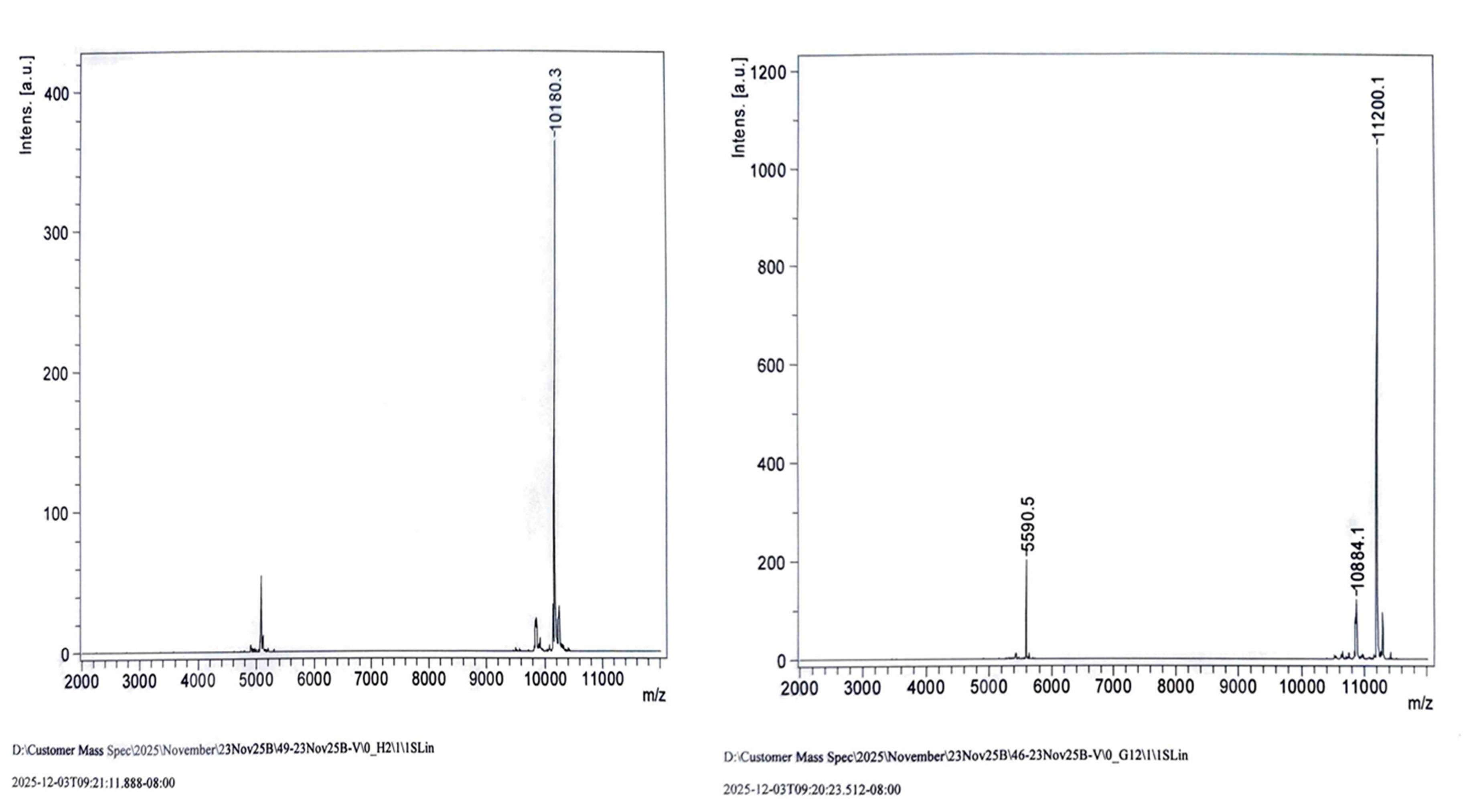

Figure S8. Mass spectrometric characterization of Vivo-Morpholino (vPMO).

Oligo Properties

Order #A126935, page 1 of 1

GENE TOOLS, LLC
Phone: (541) 929-7840
Fax: (541) 929-7841
Web: www.gene-tools.com
Email: mail@gene-tools.com
Mail: 1001 Summerton Way
Philomath, OR 97370 USA

Definitions

MW = Formula mass of Morpholino Oligo including modifications
Molar Abs. = Molar absorptivity at 265 nm in 0.1 N HCl
Weight = Delivered mass in mg
OD Units = Delivered OD (265, pH1) units
Holdback = Amount of oligo stored at GENE TOOLS for re-order

Purification: Oligos are filtered, selectively precipitated, resuspended in pure water, and freeze dried to remove all contaminants. No acids or salts used in purification.
Handling, standard Morpholino: Dissolve in sterile water to 1 mM. You may autoclave to disrupt molecular aggregates or sterilize.
Handling, Vivo-Morpholino: Dissolve in sterile water to 0.5 mM. You may heat to 65°C to disrupt molecular aggregates.
Storage: Store stock solution at room temperature in humid environment. Keep fluorescent-tagged oligos in a dim or dark place.

Your shipment may include additional information sheets if your oligos feature certain options or modifications. If present, please refer to these for further instructions. Please note that the molecular weight shown below includes any options.

Name/Quantity	Sequence/Oligo Options/Production #	MW	Molar Abs	OD	Weight
F-vivoEx10PMO2IL1RL2RN10A<br>400 nmol	AAGATCTTCAACACCACTGTGTCCA<br>46-23Nov25B-V-K-5F	11201.05	261540	104.62	4.48
vivoEx10PMO2IL1RL2RN10A<br>2000 nmol	AAGATCTTCAACACCACTGTGTCCA<br>49-23Nov25B-V-6	10182.05	261540	523.08	20.36

Figure S9. Oligonucleotides' properties.

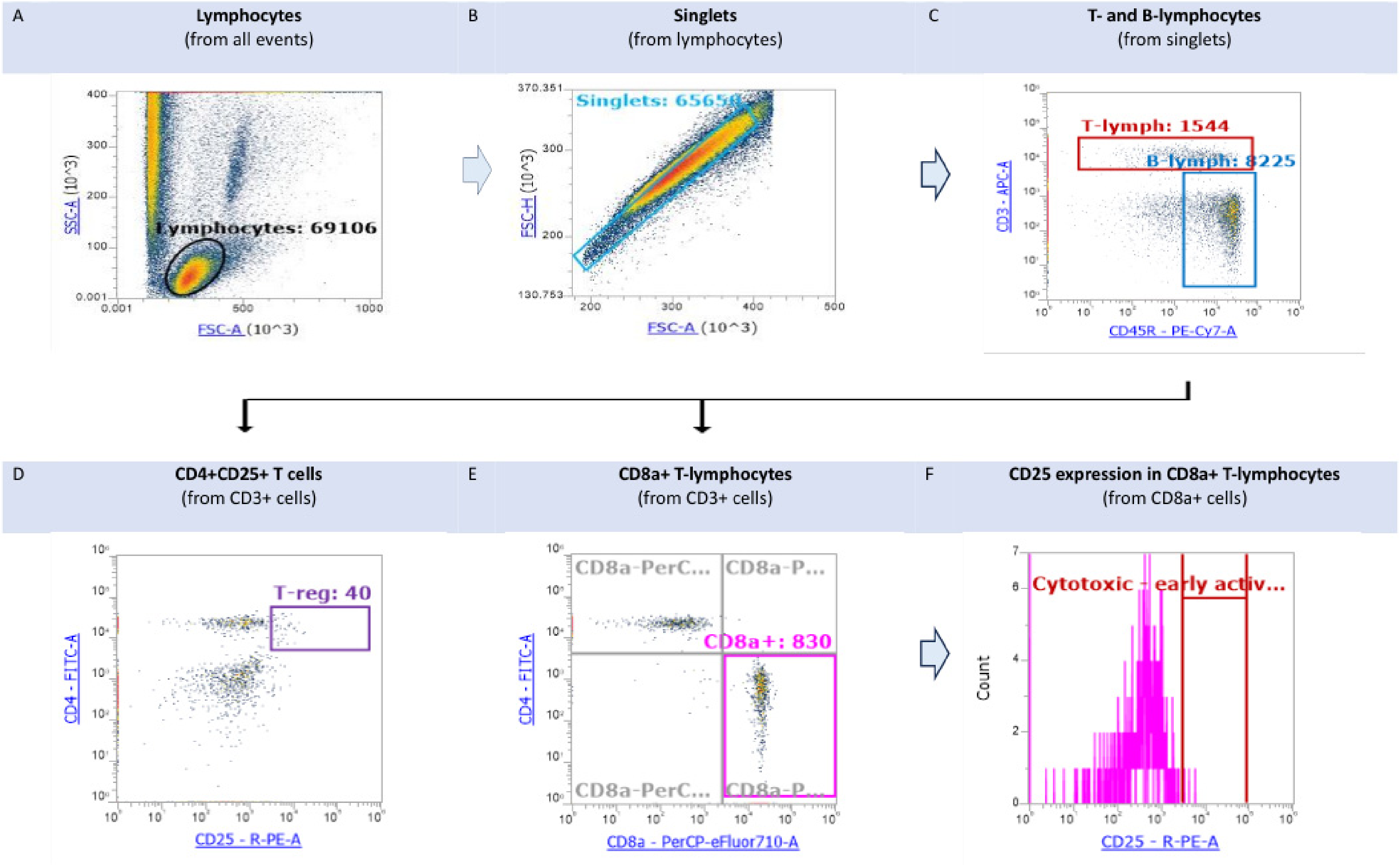

Figure S10. Flow Cytometry Gating Strategy.
